# Optimization of response surface methodology for the extraction of isoliquiritigenin from *Aspergillus niger* solid-state fermentation of licorice and its antitumor effects

**DOI:** 10.3389/fphar.2025.1629167

**Published:** 2025-11-11

**Authors:** Jingwei Hao, Qiuxuan Li, Nan Dong, Yi Zhou, Yifan Sun, Yingying Pei, Xiangkun Zhou, Lei Yang

**Affiliations:** 1 School of Life Science and Technology, Mudanjiang Normal University, Mudanjiang, China; 2 Department of Pediatrics, The Second Affiliated Hospital of Mudanjiang Medical University, Mudanjiang, China

**Keywords:** licorice, isoliquiritigenin, solid-state fermentation of *Aspergillus niger*, antitumor, intestinal flora

## Abstract

This research focused on optimizing the extraction of isoliquiritigenin (ISL) from licorice via a solid-state fermentation process involving *Aspergillus niger*. Isoliquiritigenin was quantified through high-performance liquid chromatography (HPLC), initially assessed with one-way analysis and optimized using the Box-Behnken response surface method. The extracted isoliquiritigenin underwent structural modification, and the modified derivatives with enhanced activity were screened for *in vivo* antitumor efficacy using the MTT Colorimetric (MTT) assay. Finally, the structural and compositional alterations in the intestinal flora of the mice were evaluated post-administration of the extracted isoliquiritigenin. The results indicated that the optimal extraction conditions were pH 3.7, a solid-liquid ratio of 1:2, and an *Aspergillus niger* inoculum concentration of 1.5 × 10^6^, yielding 1.53 mg/g of isoliquiritigenin after fermentation for 4 days. This yield was 9 times greater than that obtained through conventional reflux extraction and 5.46 times higher than that from the ultrasonic extraction method. Nuclear Magnetic Resonance (NMR) analysis of isoliquiritigenin (ISL) and its derivatives revealed that the ISL-b high dose group exhibited the most significant tumor suppression effect, with a suppression rate of 56.3%. In the mouse model following drug intervention, histopathological examination of kidney tissue via HE staining demonstrated that the safety profile of ISL-b was superior. Studies on the intestinal flora of mice revealed that the number of species was higher in the ISL-b high-dose group. Furthermore, there were significantly more species in the community samples than in the model group, with *Micrococcus* wartii accounting for the largest percentage at 24.49%. In the ISL-b dosing group, there was a significant increase in the abundance of Akkermansia muciniphila and Bradyrhizobium at the species level. The discoveries offer a robust scientific groundwork for developing antitumor drugs derived from isoliquiritigenin and enhance the broader application of licorice, a traditional herbal remedy.

## 1 Introduction

*Glycyrrhiza uralensis Fisch*, a perennial leguminous herb (Wang et al., 2021; Zhu et al., 2021), holds a significant position within the therapeutic framework of Traditional Chinese Medicine (TCM) (Cai et al., 2020; Yin et al., 2020). Its medicinal value is attributed to a diverse array of bioactive compounds (Nasiri et al., 2020; Ali et al., 2021), including flavonoids, polysaccharides, coumarins, inorganic elements, amino acids, organic acids, and triterpenoid saponins (Mohamad et al., 2018).

Isoliquiritigenin, a coumarin analog derived from licorice, exhibits significant antitumor activity (Chen et al., 2021; Ting and Chen, 2022). It exerts anticancer effects by inhibiting the NF-κB pathway, inducing apoptosis in cancer cells, blocking the PI3K/AKT signaling pathway, and activating ROS-mediated oxidative damage (Yu et al., 2018; Lin P.-H. et al., 2020). However, the traditional solvent extraction method is hindered by low yields (≤0.12%), the potential degradation of heat-sensitive components, and environmental pollution (Das et al., 2022; Liang et al., 2022), which severely restricts its medicinal development. In this context, microbial solid-state fermentation technology, known for its efficient biocatalysis, environmental sustainability, and product stability, has emerged as an ideal solution to overcome the bottlenecks in the industrial production of natural products (Xin et al., 2014; Wu et al., 2021). This study provides an example for the “development of microbe-derived bioactive components” in the field of natural product extraction; in the field of microbial metabolism, it lays a foundation for the subsequent investigation into the metabolic pathway of isoliquiritigenin synthesis by *Aspergillus niger* and facilitates research on microbial metabolic regulation.

*Aspergillus niger*, an engineered strain widely utilized in the production of secondary metabolites (Susca et al., 2016; Zhang et al., 2016), is a significant cellulase-producing organism (Schäpe et al., 2019; Lubna et al., 2022). It produces a comprehensive set of cellulose-degrading enzyme systems and is classified as a Generally Recognized As Safe (GRAS) strain. Its extracellular enzyme systems, including laccase and peroxidase, specifically degrade the cell wall of licorice to generate precursors of glycyrrhetinic acid, while simultaneously promoting the biosynthesis of isoglycyrrhetinic acid (Ji et al., 2022a; Wang Y. et al., 2022). However, the current fermentation process faces challenges such as significant pH fluctuations (ranging from 4.5 to 6.8) (Lima et al., 2019; Li M. et al., 2022), high temperature sensitivity (with an optimal temperature of 30 °C ± 2 °C) (Slivinski et al., 2011; Tao et al., 2022), and an imbalance in the carbon to nitrogen ratio (C/N ratio of 20–30), which leads to metabolite inhibition (Liang et al., 2021). Therefore, there is an urgent need to achieve breakthroughs in the process through a multi-objective optimization strategy.

Renal cell carcinoma (RCC) is one of the ten most common cancers worldwide and is the most prevalent type of kidney cancer (Park et al., 2020; Emaldi and Nunes-Xavier, 2022), originating from the epithelial cells of the renal tubules (Lin C.-L. et al., 2020; Kalantzakos et al., 2021). In recent years, the incidence of RCC has been steadily increasing (Scelo et al., 2018; Mittlmeier et al., 2022), with over 300,000 new diagnoses and more than 140,000 deaths attributed to this disease annually (Landberg et al., 2019; Yan L. et al., 2021). As the second leading cause of cancer-related mortality globally (Swearson et al., 2022; Wang M. et al., 2022), the resistance to chemotherapeutic drugs and the severe toxic side effects associated with malignant tumors continue to pose significant challenges in clinical treatment (Chen et al., 2019; Qian et al., 2021).

Gut microbes and hosts form a “Holobiont” (symbiotic totality) (Jiang et al., 2022; Wong et al., 2022), and their symbiosis (De Bruijn et al., 2020; Li B. et al., 2022), co-metabolism, co-evolution, and co-influence on host traits play important roles in regulating the host’s nutrient absorption, energy metabolism, immune regulation, and behavioral patterns (Ji et al., 2022b). 16S rDNA is considered the most suitable indicator for phylogenetic and taxonomic identification of bacteria (Geng et al., 2022; Zhang et al., 2022), making it the preferred nucleic acid sequence for detecting species diversity. The imbalance of intestinal flora has been shown to be closely related to tumorigenesis (Meng et al., 2021; Xie et al., 2022), development, and drug response, suggesting its significant role in antitumor research (Wang et al., 2019). Although isoliquiritigenin has demonstrated antitumor potential, its low bioavailability, short half-life, and systemic toxicity have limited its clinical application (Kong et al., 2022; Shi et al., 2022).

Based on microbial solid-state fermentation technology, this study integrates response surface optimization and the MTT method to systematically investigate the inhibitory effects of drug delivery parameters on the growth of renal cancer cells. The aim is to enhance the yield of isoglycyrrhetinic acid glycoside and to analyze the conformational relationship between its structural modifications and antitumor activity. Through the observation of cell morphology and the assessment of growth and proliferation, the study reveals the direct effects of the drug on renal cancer cells. Additionally, it incorporates 16S rDNA sequencing analysis of intestinal flora to explore the dynamic changes in the intestinal microbial community under drug intervention, establishing a multi-dimensional research system encompassing the optimization of the fermentation process, assessment of tumor cell effects, and the correlation mechanism of intestinal flora. The scientific implications of this research framework are far-reaching: For the field of natural product extraction, it provides a paradigm for “microbial fermentation + response surface optimization” to improve the yield of bound-state active components (such as the isoliquiritigenin, ISL, in licorice), effectively addressing the bottlenecks of low yield and high environmental impact in traditional extraction methods and promoting the development of green and efficient extraction technologies; for microbial metabolism research, it clarifies the role of *Aspergillus niger’s* enzyme systems (e.g., β-glucosidase) in the biotransformation of ISL (converting bound-state ISL to extractable free-state ISL), laying a solid foundation for subsequent studies on the regulation of microbial metabolic pathways related to natural product synthesis; for antitumor drug development, the screened high-activity derivative ISL-b exerts antitumor effects through dual mechanisms (direct inhibition of the NF-κB signaling pathway to induce cancer cell apoptosis + indirect regulation of intestinal flora to enhance beneficial bacteria such as Akkermansia muciniphila), offering a new target and theoretical reference for the design of natural product-derived antitumor drugs; for the modernization of Traditional Chinese Medicine (TCM), it breaks through the conventional application boundaries of licorice (Glycyrrhiza uralensis Fisch), a classic TCM herb, and expands its medicinal value to the field of antitumor research. This framework is dedicated not only to the development of an efficient and low-consumption natural product extraction process but also aims to deepen scientific knowledge regarding the antitumor effects of isoliquiritigenin from the perspective of intestinal flora, while further providing robust theoretical and technical support for the industrialization of natural antitumor drugs.

## 2 Materials and methods

### 2.1 Materials and reagents

Experimental materials: identified as Uralic Glycyrrhiza uralensis Fisch, IsoGlycyrrhiza uralensis Fisch standard (HPLC≥98%, WM256.25, C15H12O4). Hepatocellular carcinoma cells HepG-2, lung cancer cells A549, renal clear carcinoma cells Caki-1, prostate cancer PC-3 cell lines, provided by Mudanjiang Medical College.

Main reagents: Chloro-1-butyl-3-methylimidazole (Beijing Kool Chemical Technology Co., Ltd., China), Bromo-1-butyl-3-methylimidazole (Beijing Kool Chemical Technology Co., Ltd., China), 1-butyl-3-methylimidazole Hydrogen Sulfate (Beijing Kool Chemical Technology Co., Ltd., China), 1-butyl-3-methylimidazole Nitrate (Beijing Kool Chemical Technology Co., Ltd., China), Anhydrous ethanol (Nanjing Xingsha Chemical Co., Ltd., China), methanol (chromatographically pure) (Fisher Chemical, United States), acetonitrile (chromatographically pure) (Fisher Chemical, United States), acetic acid (Tenda Chemical Reagent Factory, Dongli District, Tianjin, China), hydrochloric acid (Tenda Chemical Reagent Factory, Dongli District, Tianjin, China), sodium hydroxide (Tenda Chemical Reagent Factory, Dongli District, Tianjin, China), Agar powder (Beijing Aoboshin Biotechnology Co., Ltd., China), ultrapure water (Hangzhou Wahaha Group Co., Ltd., China), yeast powder (Beijing Aoboshin Biotechnology Co., Ltd., China), RPMI1640 medium (Gibco, United States), fetal calf serum (FCS) (Zheijiang Tianhang Bio-technology Co., Ltd., China), sodium bicarbonate (NaHCO3, AR) (Tianjin Chemical Reagent Sixth Factory Branch Factory, China). Ltd., China), sodium bicarbonate (NaHCO3, AR) (Tianjin Chemical Reagent Six Factory Branch, China), sodium chloride (NaCl, AR) (Tianjin Zhiyuan Chemical Reagent Co., Ltd., China), potassium chloride (KCl, AR) (Tianjin Komeo Chemical Reagent Co., Ltd., China), disodium hydrogen phosphate (Na2HPO4-12H2O, AR) (Tianjin Tianli Chemical Reagent Co., Ltd., China), potassium dihydrogen phosphate (KH2PO4, AR) (Tianjin, China) Dongli Tianda Chemical Reagent Factory, Dongli District, China), Penicillin-Streptomycin Mixed Solution (100X Double Antibody) (Beijing Regen Biotechnology Co., Ltd., China), Tetrazolium Blue Bromide MTT (AR) (Shanghai Yuanmu Biotechnology Co., Ltd., China), PBS Buffer (Shijiazhuang Dingchen Science and Technology Co., Ltd., China), dimethyl sulfoxide DMSO (AR) (Yueyang Xiangmao Pharmaceutical and Chemical Co. Ltd., China), trypsin-EDTA culture medium (Shanghai Yuanmu Biotechnology Co., Ltd., China).

### 2.2 Instruments and equipment

Waters2695 High Performance Liquid Chromatograph (Waters, United States), Mass Spectrometry using LCQ-DecaXP/Ad type (Thermo Electron, United States), AF-08 Miniature High Speed Pulverizer (Wenling City, China, Traditional Chinese Medicine Machinery Manufacturing Co., Ltd.), DHG-9055A Electric Drum Drying Oven (Shanghai Yihang Science and Technology Co., Ltd., China), BCD-206TASJX Intelligent Refrigerator (Qingdao Haier Co., Ltd., China), BSA223S-CW Electronic Analytical Balance (Sartorius, Germany), KQ32000V Ultrasonic Cleaning Machine (Kunshan City Ultrasonic Instrument Co., Ltd., China), ZQZY-C8V Triple Combined Full-Temperature Vibrating Incubator (Shanghai Chuyi Instrument Co., Ltd., China), PB Ltd., PB-10 pH meter (Sartorius, Germany), HiQ SiL C18V column (KYA TECH), ALLEGRA-64R Beckman tabletop centrifuge (Beckman, United States), CKX53 inverted microscope (OLYMPUS), 61M/SG-XSB-102B electron microscope (China Beijing Zhongxi-Yuanda Technology Co. Ltd.), DHP-9052 Constant Temperature Incubator (Shanghai Baidian Instrument Factory, China), YDS-50 Liquid Nitrogen Storage Tank (Changsha Mingjie Instrument Co., Ltd., China), DNM-9602G Enzyme Labeling Instrument (Beijing Plan New Technology Co., Ltd., China), Micropipettes (Gilson, France), ESCOAC2-4S1 Biological Safety Cabinet (ESCO, Singapore), INC153 CO2 Incubator (Memmer, Germany), SX-700 Autoclave (Tomy, Japan), Hematocrit Plate (Shanghai Precision, China), 20∼200uL Pipette Gun (Thermo, United States), 96-well Cell Culture Plate (Corning).

## 3 Experimental methods

### 3.1 Methodological validation

Examination of exclusivity: A sample solution was prepared, and the elution solvent was used as a blank control solution. Following the determination of the standard curve, the sample was injected, and the peak area was measured to assess the method’s exclusivity. Precision test: Five samples of isoliquiritigenin were accurately measured, injected, and the peak area was determined based on the standard curve to evaluate the method’s precision. Repeatability test: Six sample solutions were prepared in parallel, injected, and the peak area was determined under high-performance liquid chromatography (HPLC) conditions to assess the method’s repeatability. Stability test: The same sample solution was injected at 0, 2, 4, 8, 12, and 24 h, and the peak area was measured to evaluate stability over the 0–24 h period. Spiking recovery experiment: Six aliquots of each isoliquiritigenin sample solution were taken, and a control solution of isoliquiritigenin of known quality was added to each. The spiking recoveries were calculated, and the relative standard deviation (RSD) values were determined to assess the method’s accuracy.

### 3.2 Isoliquiritigenin extraction by solid-state fermentation with *Aspergillus niger*

A total of 4 g of absolute dry weight licorice powder was weighed and placed in a 100 mL conical flask. Distilled water was added according to the material-liquid ratio, and the mixture was stirred thoroughly to create a solid fermentation medium, which was then sterilized. The effects of different pH values on the yield of isoliquiritigenin from licorice species were examined at pH levels of 3, 4, 5, 6, and 7. Additionally, to assess the impact of varying fermentation durations on isoliquiritigenin yield, incubation times of 2, 4, 6, and 8 days were employed. The influence of different material-liquid ratios on isoliquiritigenin yield was also investigated, with ratios of 1:1, 1:2, 1:3, 1:4, 1:5, and 1:6 being tested. Furthermore, the effect of varying inoculation amounts of *Aspergillus niger* on isoliquiritigenin yield was analyzed, with inoculation amounts of 1 × 10^5^, 5 × 10^5^, 1 × 10^6^, 2 × 10^6^, 3 × 10^6^, 4 × 10^6^, 5 × 10^6^ used. Following fermentation, 100 mL of a 75% ethanol solution was added to the medium at a 1:25 material-liquid ratio, mixed thoroughly, and extracted using ultrasonic waves in a water bath at 60 °C for 1.5 h. The extraction was performed while hot, and the resulting filtrate was collected and its volume measured.

### 3.3 Response surface experimental optimization

After the univariate study, the optimal conditions for each variable were determined, with pH, solid-liquid ratio, and *Aspergillus niger* inoculum as the independent variables, and the yield of isoliquiritigenin fractions as the dependent variable, while keeping the other factors consistent in accordance with the principles of the Response Surface Methodology (RSM) design (Table 1).

**TABLE 1:** Factors and levels.

Factor	Level		
	−1	0	1
A pH	3	4	5
BSolid-liquid ratio (g/mL)	1:1	1:2	1:3
CInoculation amount of *A. niger* (CFU/mL)	5.0 × 10^5^	1.0 × 10^6^	2.0 × 10^6^

### 3.4 Traditional extraction methods of isoliquiritigenin

Ethanol reflux extraction method: Weigh 4 g of absolute dry licorice in a 250 mL round-bottom flask. Following a 1:25 material-to-liquid ratio, add 100 mL of a 75% ethanol solution, mix thoroughly, and perform reflux extraction at 60 °C in a water bath for 1.5 h. Filter the mixture while hot and collect the filtrate.

Ultrasonic extraction: Weigh 4 g of absolute dry licorice in a 250 mL round-bottom flask. Using the same 1:25 material-to-liquid ratio, add 100 mL of a 75% ethanol solution, mix well, and extract using ultrasonication at 60 °C in a water bath for 1.5 h. Filter the mixture while hot and collect the filtrate.

### 3.5 Preparation of isoliquiritigenin derivatives

**FIGURE 1:**
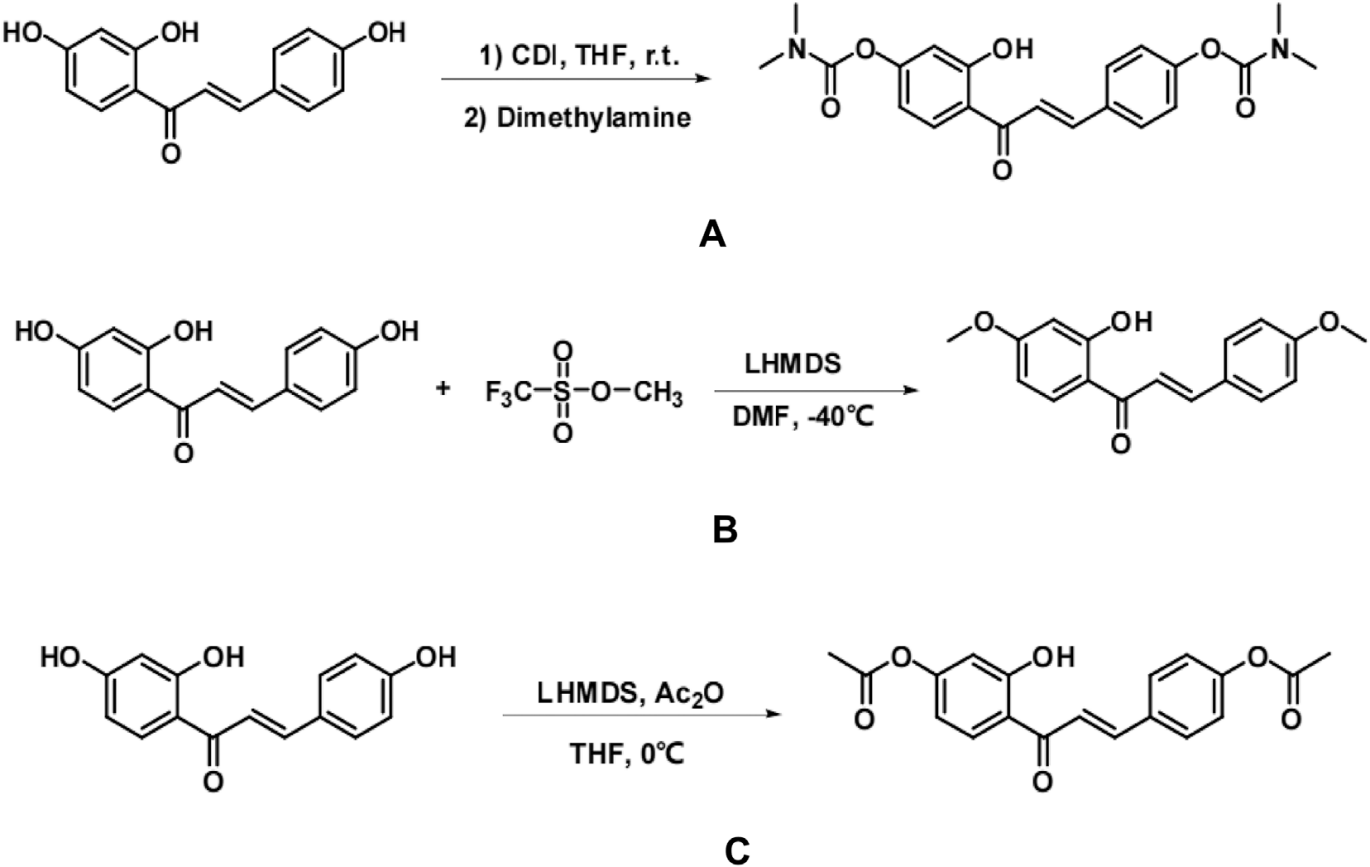
A synthetic process for the preparation of three isoliquiritigenin derivatives with isoliquiritigenin as the lead compound **(A,B,C)**.

isoliquiritigenin (200 mg, 0.78 mmol) was dissolved in 10 mL of tetrahydrofuran (THF). To the reaction solution, N,N′-carbonyldiimidazole (506 mg, 3.12 mmol) and two drops of triethylamine were added at room temperature, and the mixture was stirred for 4 h. Following this, a 30% aqueous dimethylamine solution (1.2 mL, 3.9 mmol) was introduced, and the reaction was monitored using thin-layer chromatography (TLC). Upon completion of the reaction, 40 mL of saturated aqueous ammonium chloride solution was added to quench the reaction. The aqueous phase was extracted multiple times with ethyl acetate (20 mL × 3), and the organic phases were combined. Subsequently, the organic phase was washed with saturated saline (5 mL × 2) and dried over anhydrous magnesium sulfate. After filtration and concentration, the yellow solid was obtained via column chromatography (200–300 mesh silica gel, PE/EA = 1:2) (Figure 1A).

Isolation of isoliquiritigenin (200 mg, 0.78 mmol) was achieved by dissolving it in 10 mL of N,N′-dimethylformamide (DMF). A 1.0M solution of lithium bis(trimethylsilyl) sulfamate (LHMDS) in tetrahydrofuran (1.17 mL, 1.17 mmol) was added dropwise to the reaction mixture at −40 °C under nitrogen atmosphere, and the mixture was stirred for 15 min. Subsequently, methyl trifluoromethanesulfonate (0.18 mL, 1.56 mmol) was added dropwise to the reaction solution, with the reaction progress monitored using thin-layer chromatography (TLC). Upon completion, the reaction was quenched by the addition of 40 mL of saturated aqueous ammonium chloride solution. The aqueous phases were extracted several times with ethyl acetate (20 mL × 3), and the organic phases were combined. The combined organic phase was washed with saturated saline (5 mL × 2) and dried over anhydrous magnesium sulfate. The concentrated solution was then filtered and purified by column chromatography (200–300 mesh silica gel, PE/EA = 6:1), yielding a yellow solid (Figure 1B).

isoliquiritigenin (200 mg, 0.78 mmol) was dissolved in 10 mL of THF. A 1.0M lithium bis(trimethylsilyl) diamine (LHMDS) solution in tetrahydrofuran (1.17 mL, 1.17 mmol) was slowly added dropwise to the reaction mixture under nitrogen protection at 0 °C. The reaction was stirred for 15 min, after which acetic anhydride was added dropwise to the reaction mixture (0.15 mL, 1.56 mmol). The progress of the reaction was monitored by TLC, and upon completion, 40 mL of saturated aqueous ammonium chloride solution was added to quench the reaction. The aqueous phase was extracted with ethyl acetate (20 mL × 3), and the organic phases were combined, washed with saturated saline (5 mL × 2), and dried over anhydrous magnesium sulfate. The mixture was then filtered and concentrated, followed by separation on a column (200–300 mesh silica gel, PE/EA = 1:1) to yield a yellow solid. The isoliquiritigenin derivative 3 was successfully prepared, and the synthetic process is depicted in (Figure 1C).

Finally, the derivative yields of the three substances were calculated. Calculation of derivative yields is shown in Equation 1.

$$\text{Yield }\left(\%\right)=\frac{\text{Actual}\;\text{yield}}{\text{Theoretical}\;\text{yield}}\times 100\;\%$$ (1)

The 1H-NMR spectrum and 13C-NMR spectrum were also determined by NMR using deuterated chloroform as solvent and TMS as internal standard.

### 3.6 Inhibitory effect of isoliquiritigenin and its derivatives on tumor cell proliferation detected by MTT assay

Cells were identified through observation and subsequently prepared as a cell suspension. The cell count was determined using a cell counting method, and the suspension was distributed into 96-well culture plates, with each well containing approximately 10^4^ cells in 100 μL. The cells in the 96-well plates were cultured overnight at 37 °C in a 5% CO_2_ incubator. After 24 h, the culture solution was aspirated and discarded. The final concentrations of isoliquiritigenin and its derivatives in the 96-well plates were set at 6.25 μmol/L, 12.5 μmol/L, 25 μmol/L, 50 μmol/L, and 100 μmol/L. The blank control group received an equal volume of the culture solution, which consisted of RPMI 1640 medium containing 10% fetal bovine serum. This group was cultured synchronously with the administration groups and the positive control group (37 °C, 5% CO_2_ incubator) for 48 h. It underwent the same MTT staining procedure (200 µL MTT solution added to each well for 4 h) and subsequent DMSO dissolution steps. The absorbance value from this group was used as the baseline to correct for interference from medium components, reagents, and operational processes on the experimental results. Each treatment group consisted of six parallel wells, and the cells were incubated at 37 °C in a 5% CO_2_ incubator for an additional 48 h. Following this incubation, the supernatant was removed, and 200 μL of fresh culture solution was added to each well. Subsequently, 200 μL of MTT solution was added to each well, and incubation continued for 4 h. After this period, the supernatant was discarded, ensuring minimal liquid residue remained, and 100 μL of DMSO was added and mixed thoroughly by shaking for 10 min. The cell inhibition rate was calculated based on the absorbance values obtained using an enzyme labeling instrument at a wavelength of 490 nm, and is shown in Equation 2.

$$\text{Cell proliferation rate }\%=\frac{\text{Mean}\;\text{OD}\;\text{of}\;\text{administered}\;\text{group}-\text{Mean}\;\text{OD}\;\text{of}\;\text{blank}\;\text{control}\;\text{group}}{\text{Mean}\;\text{OD}\;\text{value}\;\text{of}\;\text{blank}\;\text{control}\;\text{group}}\times 100\;\%$$ (2)

### 3.7 Establishment of mouse tumor models

Caki-1 cells were cultured and suspended in serum-free medium, adjusting the cell concentration to 2 × 10^6^ cells/mL. Under aseptic conditions, the right anterior axillary subcutis and abdomen of the mice were sterilized using 75% ethanol, followed by the inoculation of 0.2 mL of the cell suspension per mouse, ensuring thorough mixing before each aspiration to establish a solid tumor model. Tumor volume was measured every 3 days. The mice were randomly divided into five groups, each containing ten mice, and received intraperitoneal (ip) administration post-inoculation. The experimental groups were administered ISL-bL at high, medium, and low doses of 50 mg/(kg·d), 25 mg/(kg·d), and 12.5 mg/(kg·d), respectively. The positive control group received IL-2 at a dose of 2.5 × 10^5^ IU/(kg·d), while the model control group was given an equal volume of saline. Administration occurred once daily, with 0.2 mL per mouse, starting the day after inoculation. The animal room was well-ventilated, maintained at a temperature of 20 °C–22 °C and a humidity of 45%–50%. The environment was clean, quiet, and followed a natural circadian rhythm. The animals were fed a standard diet, equivalent to 3 g per mouse per day, with water provided continuously. Fresh drinking water was replaced daily, and bedding was changed every 2–3 days.

### 3.8 HE staining to observe the morphological changes of tumor tissues

Tumor tissues of appropriate size were fixed in a 4% paraformaldehyde solution, dehydrated, and embedded in clear paraffin. The samples were then sealed in neutral gel through a series of processes, including dewaxing, gradient ethanol dehydration, differentiation rinsing, and HE staining, after which they were placed under a microscope for observation. Finally, statistical analyses were performed using SPSS 18.0 and GraphPad Prism 6.01 software, with data from each group expressed as x ± s. Sample comparisons were conducted using a one-way ANOVA test, and a P-value of less than 0.05 was considered statistically significant.

### 3.9 Determination of the effect of ISL-b on protein expression in Caki-1 cells by Western blot method

The effect of ISL-b on protein expression in Caki-1 cells was determined by Western blot. The protein samples were thawed at room temperature, processed and denatured. Then, gel preparation and electrophoresis were performed, followed by membrane transfer in sequence. The membranes were blocked overnight with 5% milk prepared in TBST, incubated with primary and secondary antibodies successively with washing steps in between. Finally, ECL color development was carried out in the dark, and imaging analysis was performed.

### 3.10 Intestinal bacteria group-metabolic mechanisms in the Caki-1 homozygous mouse model using 16SrRNA sequencing

On the first day following the completion of modeling, rat feces were aseptically collected into sterile EP tubes. For both the model group and the IL-2 positive control group, five mice were randomly selected, and their feces were pooled. Two samples were taken from each of the ISL groups categorized by low, medium, and high doses. Each sample consisted of feces from five randomly selected mice, from which 200 mg was extracted. The collected fresh feces were snap-frozen in liquid nitrogen for over 30 s and subsequently stored at −80 °C for future use. DNA extraction, amplification, and library construction were completed within 4 weeks to facilitate subsequent analysis.

The specific sequencing and analysis workflow is as follows: Illumina NovaSeq platform was used for paired-end sequencing (PE250). The sequencing depth was evaluated by rarefaction curves, and the sequencing depth was determined to be reasonable when the curves tended to be flat. Prior to alpha diversity analysis, normalization was performed using a minimum data volume threshold of 59,860. For data standardization, the minimum sample size normalization strategy was adopted, involving random sampling adjustment of effective sequences from all samples. For bioinformatics analysis, QIIME v1.9.1 was used for Tags quality control and alpha diversity index calculation; Uparse v7.0.1001 was used for OTU clustering with 97% identity. Additionally, FLASH v1.2.7 was used for sequence assembly, Vsearch for chimera filtering, and MUSCLE v3.8.31 for constructing phylogenetic trees. The complete workflow was as follows: raw off-machine data were assembled into raw Tags by FLASH, then subjected to QIIME quality filtering to obtain clean Tags. After removing chimeras via Vsearch, effective sequences were obtained, which were subsequently used for OTU clustering, species annotation, and diversity analysis. The quality filtering criteria included truncating consecutive 3 or more bases with a quality score ≤19 and removing Tags with a length less than 75% of the original sequence.

## 4 Results and analysis

### 4.1 Methodology establishment

#### 4.1.1 Linear relationships

**FIGURE 2:**
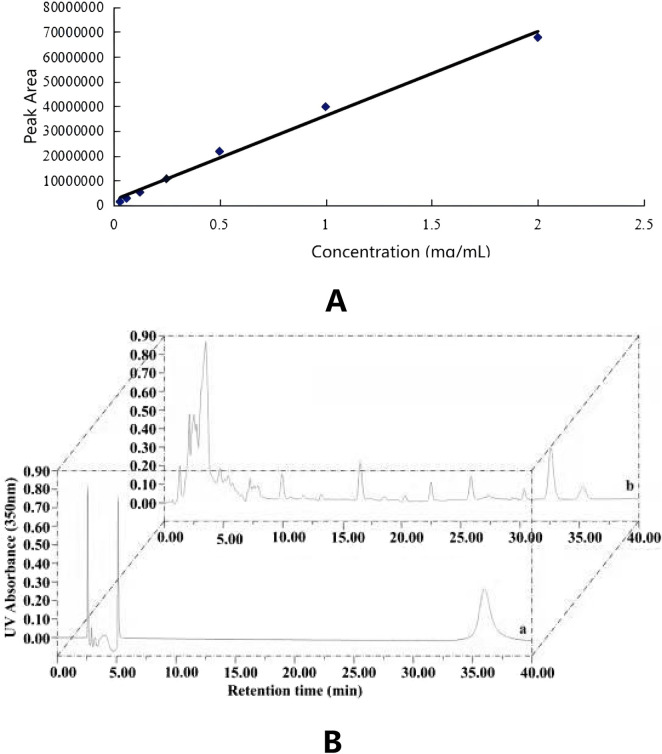
HPLC chromatogram for isoliquiritigenin in standard solutions **(A)** and sample extract **(B)**.

The HPLC conditions involved using a C18 column (4.6 × 250 mm, 5 μm) with UV detection at a wavelength of 350 nm. The mobile phase was composed of acetonitrile mixed with 0.5% aqueous acetic acid in a volume ratio of 32:68.5 (v/v). The injection volume was 10 μL, with a flow rate of 1.0 mL/min, and the column temperature was maintained at 25 °C. The isoliquiritigenin standard was prepared in seven concentration gradients: 0.03125, 0.06250, 0.12500, 0.25000, 0.50000, 1.00000, and 2.00000 mg/mL, and analyzed using the aforementioned mobile phases. The peak area of isoliquiritigenin was measured by absorbance at 350 nm. Subsequently, a standard curve was constructed with the concentration of the isoliquiritigenin standard (X) plotted on the horizontal axis and the peak area (Y) on the vertical axis, leading to the derivation of the regression equation, with the resulting high-performance liquid chromatogram presented in Figure 2A. Additionally, Figure 2B illustrates the standard curve for isoliquiritigenin, yielding the regression equation y = 3 × 10^7^ x + 2 × 10^6^ and a correlation coefficient of *R* ^2^ = 0.9911. A strong linear relationship between concentration and absorbance was observed within the range of 0–2.0 mg/mL.

#### 4.1.2 Methodological examination

Exclusivity test: The experimental results demonstrated that the blank test solution exhibited minimal absorbance under the chromatographic conditions, with no interference observed in the chromatographic analysis of the test material. This indicates that the established method possesses good exclusivity. Precision experiment: The RSD value is 0.42%, indicating that the precision of this method is satisfactory (Tables 2–5).

**TABLE 2:** Precision experiment results.

Content (mg/g)	Mean value (mg/g)	RSD (%)
0.413	0.416	0.420
0.415		
0.416		
0.417		
0.418		
0.415		

**TABLE 3:** Results of stability experiments.

Time (h)	Content (mg/g)	Mean value (mg/g)	RSD (%)
0	0.413	0.413	0.560
2	0.410		
4	0.414		
8	0.411		
12	0.416		
24	0.415		

**TABLE 4:** Repeatability experiment results.

Content (mg/g)	Mean value (mg/g)	RSD (%)
0.413	0.412	1.23
0.405		
0.414		
0.412		
0.419		
0.407		

**TABLE 5:** Experimental results of sample recovery.

Content (mg)	Amount of control added (mg)	Measured quantity (mg)	Recovery rate (%)	Average (%)	RSD (%)
1.02	1.01	1.99	98.03	98.20	0.52
1.00	1.02	1.98	98.02		
1.01	1.03	1.99	97.54		
1.06	1.00	2.03	98.54		
1.04	1.02	2.02	98.05		
1.05	1.01	2.04	99.02		

### 4.2 Optimization of isoliquiritigenin extraction by solid-state fermentation of *Aspergillus niger*

#### 4.2.1 Effect of fermentation pH on the yield of isoliquiritigenin

**FIGURE 3:**
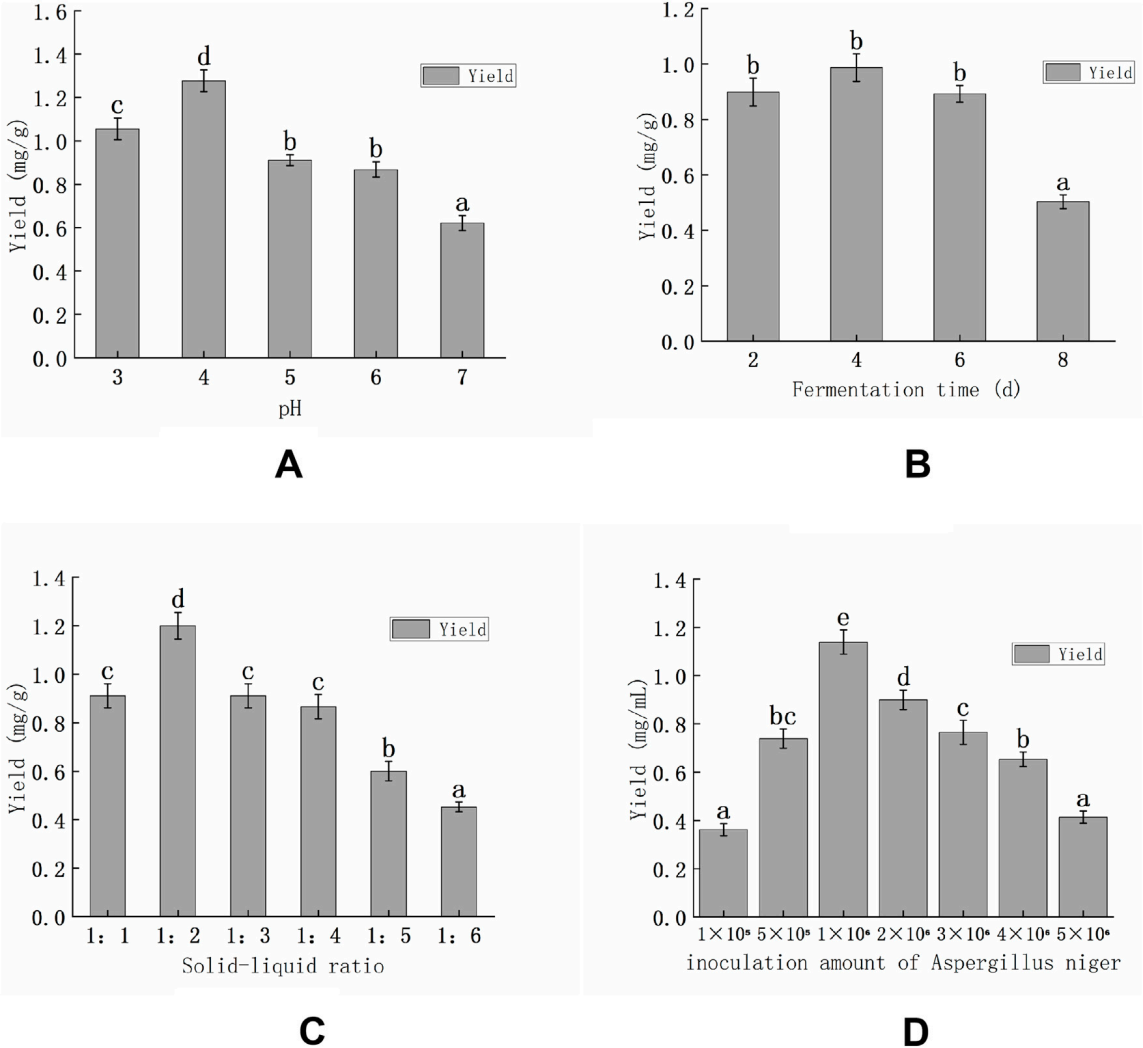
Effect of fermentation pH on isoliquiritigenin yield **(A)**; Effect of fermentation time on isoliquiritigenin yield **(B)**; Effect of fermentation solid-liquid ratio on isoliquiritigenin yield **(C)**; D Effect of *Aspergillus niger* inoculum on isoliquiritigenin yield **(D)**. (Note: Identical letters on the bar graph indicate a non-significant difference, while differing letters signify a significant difference between treatments) (P < 0.05). In the figure, a, b, c, d are symbols labeled after statistical analysis (such as multiple comparisons), used to characterize the difference conditions among different groups. Groups labeled with the same letter show no significant difference in the index, while groups labeled with different letters show significant differences.

The effect of fermentation pH on the yield of isoliquiritigenin from licorice is illustrated (Figure 3A). The yield of isoliquiritigenin was maximized at a pH of 4 and exhibited variability with differing pH levels. Specifically, the yield of isoliquiritigenin decreased as fermentation pH increased.

#### 4.2.2 Effect of fermentation time on the yield of isoliquiritigenin

The effect of fermentation time on the yield of isoliquiritigenin was investigated. As illustrated in Figure 3B, the yield of isoliquiritigenin increased with an extended fermentation period, reaching its peak at 4 days.

#### 4.2.3 Effect of fermentation solid-liquid ratio on the yield of isoliquiritigenin

The feed-liquid ratio significantly influences the yield of isoliquiritigenin extracted from licorice. The yield of isoliquiritigenin decreases with an increasing material-liquid ratio (Figure 3C). The optimal yield of isoliquiritigenin was achieved at a material-liquid ratio of 1:2.

#### 4.2.4 Effect of *Aspergillus niger* inoculum concentration on the extraction of isoliquiritigenin from glycyrrhiza uralensis fisch

The yield of isoliquiritigenin exhibited a linear increase with the rising concentration of *Aspergillus niger*, reaching its maximum yield at an inoculum concentration of 1.0 × 10^6^ CFU/mL (Figure 3D). The mycelial biomass also peaked at the same inoculum concentration, indicating that the enzyme secretion achieved an optimal ratio with the substrate concentration (isoglycoside), thereby maximizing the efficiency of the catalytic reaction per unit time. Experimental data revealed that the yield was enhanced by approximately 65% when the inoculum concentration was raised from 1.0 × 10^5^ to 1.0 × 10^6^, which aligns with the kinetic principle that the enzyme reaction rate increases linearly with enzyme concentration (Alves et al., 2018; Alarid-García et al., 2022).

### 4.3 Response surface method optimization

The interactions among these factors were investigated using Response Surface Methodology (RSM) to optimize fermentation pH, solid-liquid ratio, and the inoculum of *Aspergillus niger*. The experiments were randomized, and were designed to maximize the impact of unexplained variability on extraction efficiency. A total of 17 tests were conducted with 5 replications (specifically the 2nd, 7th, 8th, 12th, and 15th tests) to accurately estimate the pure sum of squares of errors (Table 6).

**TABLE 6:** Box-Behnken experimental design.

Run	Factor A	Factor B	Factor C
	pH	Solid-liquid ratio (g/mL)	Inoculation amount of *A. niger* (CFU/mL)
1	5	3	1.25 × 10^6^
2	4	2	1.25 × 10^6^
3	4	1	5.00 × 10^5^
4	3	1	1.25 × 10^6^
5	4	3	5.00 × 10^5^
6	5	2	2.00 × 10^6^
7	4	2	1.25 × 10^6^
8	4	2	1.25 × 10^6^
9	3	2	2.00 × 10^6^
10	5	2	5.00 × 10^6^
11	4	3	2.00 × 10^6^
12	4	2	1.25 × 10^6^
13	3	3	1.25 × 10^6^
14	4	1	2.00 × 10^6^
15	4	2	1.25 × 10^6^
16	5	1	1.25 × 10^6^
17	3	2	5.00 × 10^5^

Based on the data presented, a multiple regression fitting analysis was conducted using Design Expert 8.0.7.1 software. The resulting quadratic equation for the yield of isoglycosides (Y) in relation to each factor variable is as follows:Y% = 1.49–0.016A + 0.054B + 0.20C - 0.095AB + 0.0025AC - 0.062BC - 0.049A^2^ - 0.21B^2^ - 0.33C^2^. The ANOVA results, indicate that the model is statistically significant with a p-value of less than 0.0001. Furthermore, the misfit term F-value of 33.51 suggests that the misfit is not significant (Table 7).

**TABLE 7:** Analysis of variance (ANOVA) for regression equations.

Source	Sum of squares	Df	Mean square	F-value	*P*-value
Model	1.1100	9	0.1200	33.5100	<0.0001
A-pH	2.1120 × 10^−3^	1	2.1120 × 10^−3^	0.5700	0.4739
B-solid liquid ratio	0.0230	1	0.0230	6.2600	0.0408
C-inoculation amount of *A. niger*	0.3300	1	0.3300	88.9100	<0.0001
AB	0.0360	1	0.0360	9.7800	0.0167
AC	2.5000 × 10^−5^	1	2.5000 × 10^−5^	6.7760 × 10^−3^	0.9367
BC	0.0160	1	0.0160	4.2300	0.0786
A2	0.0100	1	0.0100	2.7800	0.1391
B2	0.1800	1	0.1800	50.0400	0.0002
C2	0.4600	1	0.4600	125.7100	<0.0001
Residual	0.0260	7	3.6900 × 10^−3^		
Lack of Fit	0.0110	3	3.7080 × 10^−3^	1.0100	0.4759
Pure Error	0.0150	4	3.6760 × 10^−3^		
Cor Total	1.1400	16			

**FIGURE 4:**
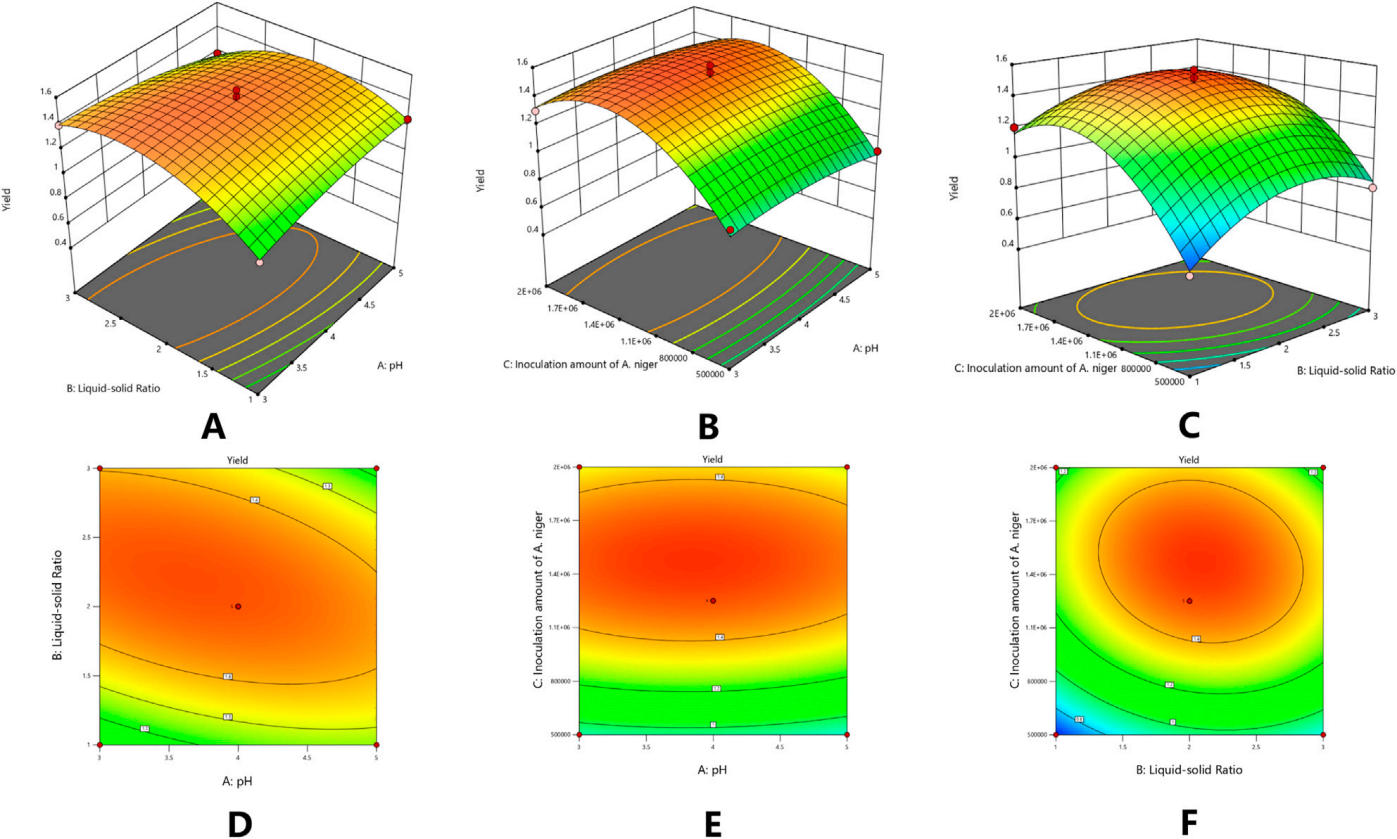
Response surface plots (3-D) of concentration of [BMIM] Br and extraction time on the yield of isoliquiritigenin **(A)**; Response surface plots (3-D) of concentration of [BMIM] Br and solid-liquid ratio on the yield of isoliquiritigenin **(B)**; Response surface plots (3-D) of extraction time and solid-liquid ratio on the yield of isoliquiritigenin **(C-F)**.

Based on the credibility analysis of the regression equation presented, the model’s adjusted coefficient of determination is *R* ^2^_adj = 0.9482, indicating that this model can explain 94.82% of the variation in the response variable. Additionally, the coefficient of determination is *R* ^2^ = 0.9773 and the predicted coefficient of determination is *R* ^2^_pred = 0.8235, with a precision ratio of 18.461. This precision ratio suggests that the model is sufficiently precise, and the goodness of fit results are effective for predicting the yield of isoGlycyrrhiza uralensis Fisch under varying conditions for the yield of isoliquiritigenin. Furthermore, based on the comparison of F-values, it can be concluded that the order of significance under different conditions is as follows: *Aspergillus niger* inoculum > solid-liquid ratio > pH value (Table 8) (Figures 4A–F).

**TABLE 8:** The credibility analysis of the regression equations.

Index marka	Extraction efficiency of lignans
Std. Dev	0.0610
Mean	1.2100
C.V. %	5.0200
PRESS	0.2000
R-Squared	0.9773
AdjR-Squared	0.9482
PredR-Squared	0.8235
Adeq Precision	18.4610

The optimal combination obtained from the response surface modeling calculation, along with the results analyzed and estimated using prediction software, yielded a pH of 3.694, a solid-liquid ratio of 1:2.155, an *Aspergillus niger* inoculum concentration of 1,466,745, and a yield of 1.525 mg/g. To enhance practical operational convenience, the extraction process parameters were adjusted to pH 3.694, a solid-liquid ratio of 1:2.155, and an *Aspergillus niger* inoculum concentration of 1,466,745 for three validation tests. The validation analysis produced a yield of 1.49 ± 0.035 mg/g of isoliquiritigenin, demonstrating that these parameters effectively reflect the impact of screening factors on the yield of isoliquiritigenin.

### 4.4 Preparation of isoliquiritigenin derivatives and their structural characterization

#### 4.4.1 Yield determination

Among them, the theoretical yield of ISL - a was 310 mg, the actual yield was 127 mg, and the yield was 40.9%; the theoretical yield of ISL - b was 221 mg, the actual yield was 175 mg, and the yield was 79.1%; the theoretical yield of ISL - c was 265 mg, the actual yield was 229 mg, and the yield was 86.4%. These data visualize the yield and profitability of each serial number (Table 9).

**TABLE 9:** Yield of each derivative.

Serial number	Theoretical yield (mg)	Actual yield (mg)	Yield (%)
ISL-a	310	127	40.9
ISL-b	221	175	79.1
ISL-c	265	229	86.4

#### 4.4.2 Identification of target compounds

##### 4.4.2.1 Determination of chemical shifts of active hydrogens in isoglycosides

Isoliquiritigenin: ESI-MSm/z: 257.18 (M + H)+.1HNMR (400 MHz, DMSO-d6) δ13.61 (s, 1H), 10.68 (s, 1H), 10.14 (s, 1H), 8.17 (d, J = 9.0 Hz, 1H), 7.76 (m, 4H), 6.84 (d, J = 8.6 Hz, 2H), 6.41 (dd, J = 8.9, 2.3 Hz, 1H), 6.28 (d, J = 2.3 Hz, 1H). 13CNMR (101 MHz, DMSO-d6) δ 191.43, 165.68, 164.85, 160.16, 144.17, 132.76, 131.13, 125.64, 117.30, 115.73, 112.88, 107.98, 102.47.

**FIGURE 5:**
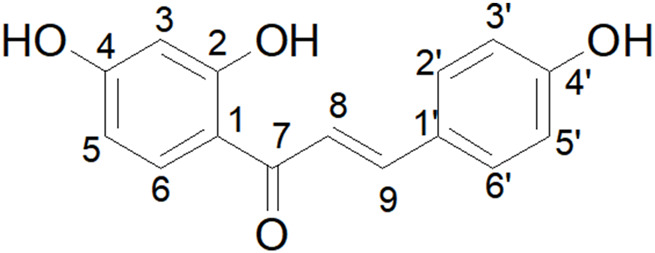
Schematic diagram of the chemical structure of isoliquiritigenin, with the numbering of carbon atoms (1–9 and 1′–6′).

Isoliquiritigenin contains three hydroxyl groups situated at the 2- and 4-positions of the A-ring and at the 4′position of the B-ring. This paper focuses on structural modifications of isoliquiritigenin derivatives, specifically targeting the hydroxyl groups. Consequently, determining the chemical shifts of the active hydrogens in isoliquiritigenin is essential. Deuterated DMSO was employed as the solvent, and the final resolved data for isoliquiritigenin were obtained through analysis of the 1H-NMR, 13C-NMR, DEPT135, HSQC, and HMBC 1D and 2D NMR spectra (Supplementary Table 1A; Supplementary Table 1B; Supplementary Table 1C) (Figure 5).

The chemical shift of the hydroxyl group at the C2 position is observed at 13.61 ppm, significantly higher than that of the hydroxyl groups at the C4 and C4′ positions. The peak shape indicates that the hydrogen signal of C2-OH is sharper, whereas the signals from the generally active hydrogens, such as those from C4-OH and C4′-OH, are broader and less defined. This phenomenon can be attributed to the formation of an intramolecular hydrogen bond between the hydroxyl group at the C2 position and H-8, which results in an increased chemical shift and a sharper peak shape.

##### 4.4.2.2 Activity of the hydroxyl group in isoliquiritigenin

Based on the experimental results presented in this paper, it is evident from the 1H-NMR spectra of compound 1 and compound 2 that the hydrogen signals corresponding to the hydroxyl groups at the C4 and C4′ positions in isoglycyrrhetinin have disappeared within the chemical shift range of δ10-11. This observation allows us to conclude that the reaction sites are located at the hydroxyl groups in the C4 and C4′ positions, which exhibit similar reactivity without a specific sequential order. In contrast, the hydroxyl group at the C2 position demonstrates increased stability and is less likely to react with other chemical reagents. This stability can be attributed to the formation of intramolecular hydrogen bonding and the influence of steric hindrance, which impede the approach of substituent groups to this site.

##### 4.4.2.3 Spectral analysis of isoliquiritigenin ISL-a

ISL-a: yellow solid 127 mg, 40.9% yield, melting point 124 °C–126 °C. ESI-MS, *m/z*: C_21_H_23_N_2_O_6_ [M + H]^+^, theoretical value 399.17, measured value 399.42. ^1^HNMR (400 MHz, CDCl3) δ 13.62 (s, 1H), 7.48 (d, J = 8.5 Hz, 2H), 7.14 (d, J = 8.5 Hz, 2H). 6.90 (d, J = 8.4 Hz, 1H), 6.72 (d, J = 2.2 Hz, 1H), 6.67 (dd, J = 8.4, 2.2 Hz, 1H), 6.08 (d, J = 3.6 Hz, 1H), 5.85 (d, J = 3.6 Hz, 1H), 2.31 (s, 3H), 2.29 (s, 3H), 2.09 (s, 6H). ISL-a 1 was identified as 4-[3-(4-dimethylaminocarbonyloxyphenyl)-acryloyl]-3-hydroxy-dimethylcarbamic acid phenyl ester (ISL-a).

##### 4.4.2.4 isoliquiritigenin ISL-b spectral analysis

ISL-b: yellow solid 175 mg, yield 79.1%, melting point 131∼132 °C. ESI-MS, *m/z*: C_17_H_17_O_4_ [M + H]^+^, theoretical value 285.11, measured value 285.20. ^1^HNMR (400 MHz, DMSO-d6) δ 13.62 (s, 1H), 8.29 (d, J = 9.0 Hz, 1H), 7.90 (dd, J = 15.4, 8.8 Hz), 7.81 (d, J = 15.4, 8.8 Hz), 7.04 (d, J = 15.4 Hz, 2H), 6.57 (dd, J = 8.8 Hz, 2H), 3H), 7.81 (d, J = 15.4 Hz, 1H), 7.04 (d, J = 8.8 Hz, 2H), 6.57 (dd, J = 9.0, 2.5 Hz, 1H), 6.52 (d, J = 2.5 Hz, 1H), 3.85 (s, 3H), 3.83 (s, 3H). ISL-b was identified as (E)-1-(2-hydroxy4-methoxyphenyl)-3-(4-methoxyphenyl)-2-propen-1-one (ISL-b).

##### 4.4.2.5 isoliquiritigenin ISL-c spectral analysis

ISL-c: yellow solid 229 mg, yield 86.4%, melting point 121∼122 °C. ESI-MS, *m/z*: C_19_H_17_O_6_ [M + H]^+^, theoretical value 341.10, measured value 341.13.^1^HNMR (400 MHz, DMSO-d6) δ 13.61 (s, 1H), 8.17 (d, J = 9.0 Hz, 1H), 7.76 (dd, J = 5.2, 3.3 Hz 4H), 6.84 (d, J = 8.6 Hz, 2H), 6.41 (dd, J = 8.9, 2.3 Hz, 1H), 6.28 (d, J = 2.3 Hz, 1H), 2.26 (s, 6H). ISL-c was identified as (E)-1-(2-hydroxy4-acetoxyphenyl)-3-(4-acetoxyphenyl)-2-propen-1-one (ISL-c).

##### 4.4.2.6 Structural confirmation conclusions

The structures of the isoliquiritigenin derivatives (ISL-a, ISL-b, ISL-c) synthesized in this study were jointly confirmed through four independent sets of experiments: one-dimensional/two-dimensional nuclear magnetic resonance (1D/2D NMR), mass spectrometry (MS), melting point determination, and HPLC purity validation. All characterizations were based on high-purity samples (≥98% purity) purified by HPLC. Method validation demonstrated precision (RSD = 0.42%), recovery (RSD = 0.52%, 98.20%), stability (RSD = 0.56%), and repeatability (RSD = 1.23%), effectively eliminating impurity interference and ensuring data reproducibility. Taking the most bioactive ISL-b (methoxy-modified derivative) as an example, the characteristic peaks in 1H-NMR include: methoxy proton peak (δ 3.85 s, 3H; δ 3.83 s, 3H), and trans double bond proton peak (δ 7.90 days, J = 15.4 Hz; δ 7.81 days, J = 15.4 Hz) are clearly defined. HSQC coupling with ^13^C-NMR confirms methoxy attachment at C4 of ring A and C4′ of ring B, while HMBC further establishes the core integrity of the “ring A - carbonyl - double bond - ring B″ skeleton. ESI-MS measured its (M + H)^+^ peak at 285.20 Da (theoretical molecular weight deviation ≤0.25 Da). The melting point (131 °C–132 °C) overlaps with the literature range for similar chalcones, ruling out isomerism or substitution site errors. The characterization rationale for ISL-a and ISL-c aligns with ISL-b. ESI-MS (399.42 Da, 341.13 Da), melting points (124 °C–126 °C, 121 °C–122 °C), and 1D/2D NMR data all match theoretical structures. Furthermore, the C4/C4′-position methoxy modification in ISL-b aligns strongly with its optimal antitumor activity (Caki-1 cell IC50 = 15.304 μmol/L, tumor inhibition rate 56.3%), The p-π conjugation effect of the methoxy group enhances its binding affinity to the p65 protein in the NF-κB pathway (Western blot analysis showed the most significant downregulation of p-p65 protein), providing structure-activity relationship evidence to support the rationality of the structural confirmation. In summary, the structural confirmation data for the three derivatives are comprehensive and logically rigorous, meeting the academic standards for structural characterization of new compounds.

### 4.5 Inhibitory effect of isoliquiritigenin and its derivatives on tumor cell proliferation and cell morphology observation

#### 4.5.1 Inhibition of tumor cell proliferation by isoglycosides and their derivatives

**FIGURE 6:**
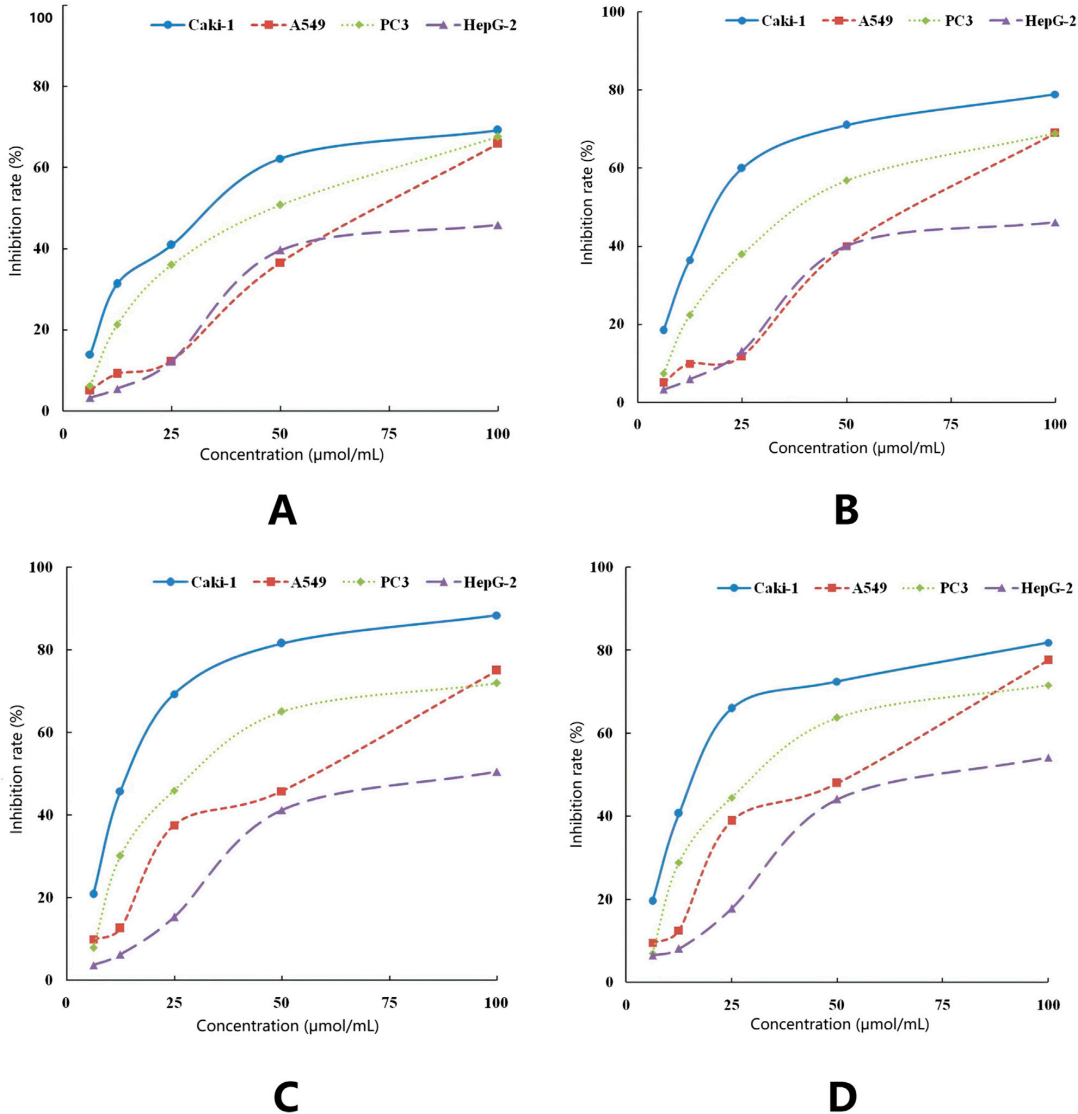
Inhibitory effect of ISL on the growth oftumorcells **(A)**, Inhibitory effect of ISL-a on the growth of tumor cells **(B)**, Inhibitory effect of ISL-b on the growth of tumor cells **(C)**, Inhibitory effect ofISL-c on the growth of tumor cells **(D)**.

The cytotoxic effects of ISL and its derivatives on hepatocellular carcinoma HepG-2, lung cancer cell line A549, renal clear cell carcinoma Caki-1, and prostate cancer cell line PC-3 were assessed using the MTT assay. The IC50 values and inhibition rates of ISL and its three derivatives were calculated, following a 48-hour treatment period (Figures 6A–D). Notably, ISL and its derivatives exhibited superior inhibitory effects on renal clear cell carcinoma Caki-1, with IC50 values of 35.087 μmol/L for ISL, 21.692 μmol/L for ISL-a, 15.304 μmol/L for ISL-b, and 18.611 μmol/L for ISL-c. As illustrated in the graphs, the isoliquiritigenin derivative ISL-b demonstrated the most effective inhibition of Caki-1 cells *in vivo* (Table 10).

**TABLE 10:** Growth inhibition of tumor cells after 48 h of action of isoglycosides and their derivatives (x ± s, n = 3).

Groups	IC50 (μmol/L)			
	Caki-1	A549	PC3	HepG-2
ISL	35.087	70.604	47.643	96.782
ISL-a	21.692	65.015	42.486	95.802
ISL-b	15.304	46.405	32.762	85.548
ISL-c	18.611	43.370	34.287	78.010

### 4.6 Morphological effects of isoliquiritigenin and derivatives on renal cancer cells (Caki-1)

**FIGURE 7:**
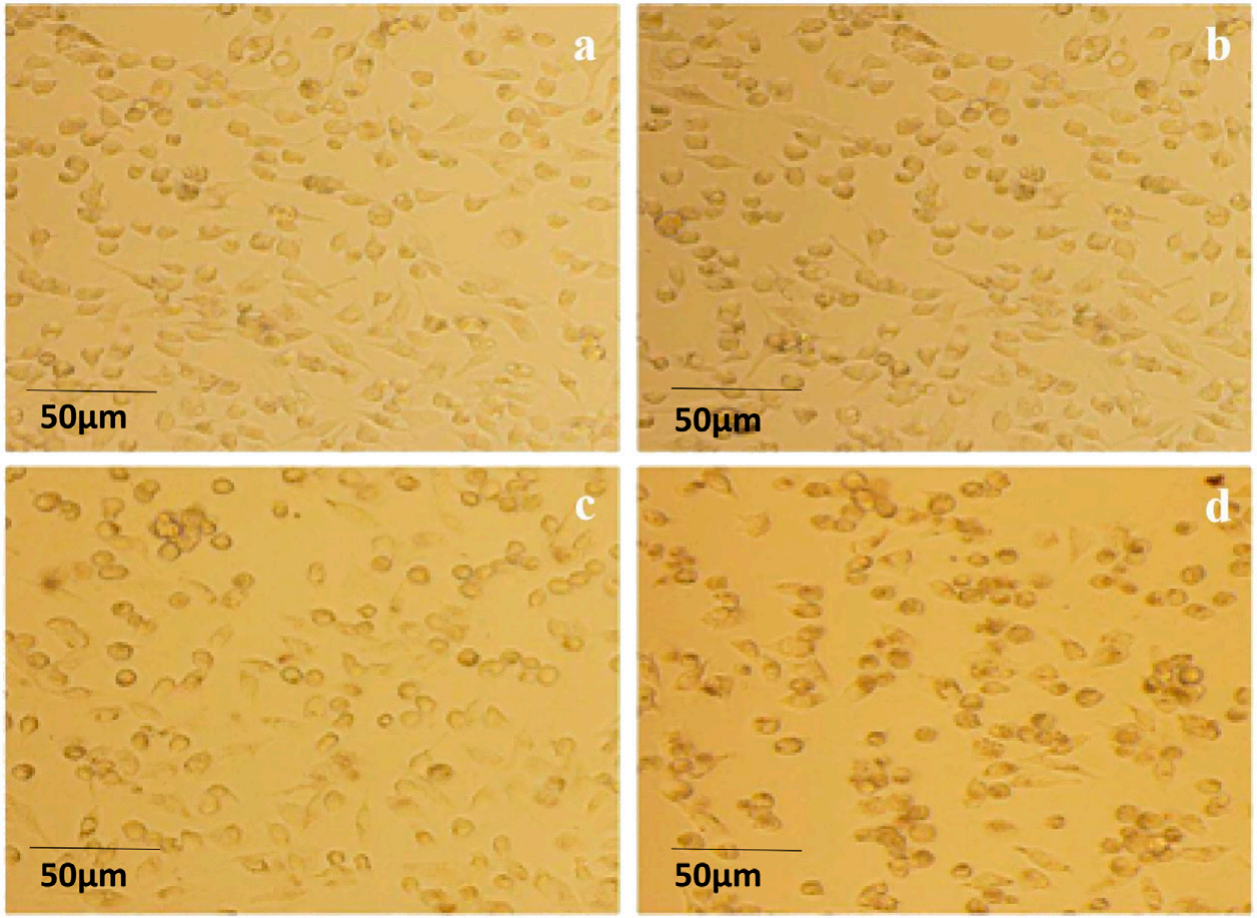
Morphological appearance of Caki-1 cells by optical microscope (200×) **(a)** ISL; **(b)** 25 μg/mLISL-a; **(c)** 25 μg/mL ISL-c; **(d)** 25 μg/mL ISL-b.

Cell morphology was observed under an inverted microscope after treatment with ISL and its derivatives on Caki-1 cells at a concentration of 25 μmol/L for 24 h. After 24 h of ISL treatment, most Caki-1 cells appeared to be in good condition, exhibiting adherent growth and well-defined edges; however, a few cells exhibited a rounded morphology. In the presence of the ISL-a compound, the number of rounded cells increased compared to the ISL-treated group, with non-adherent cells becoming evident and sparse growth observed, indicating that most cells were suspended in the culture medium. With the addition of the ISL-c compound, the number of rounded and non-adherent cells further increased, while the growth rate of adherent cells significantly decreased. In the ISL-b treatment group, the morphological changes in Caki-1 cells were more pronounced compared to the ISL group; most cells became rounded, and the transparency and fullness of the cells decreased, with some cells appearing ruptured and a majority exhibiting signs of cell death. These results indicate that all three derivatives had a more pronounced inhibitory effect on Caki-1 cells than ISL, with ISL-b demonstrating the most significant efficacy (Figure 7).

### 4.7 Effects of isoglycosides and derivatives on Caki-1 hormonal mice

#### 4.7.1 Effect of isoliquiritigenin and its derivatives on tumor volume changes in Caki-1 loaded mice

**FIGURE 8:**
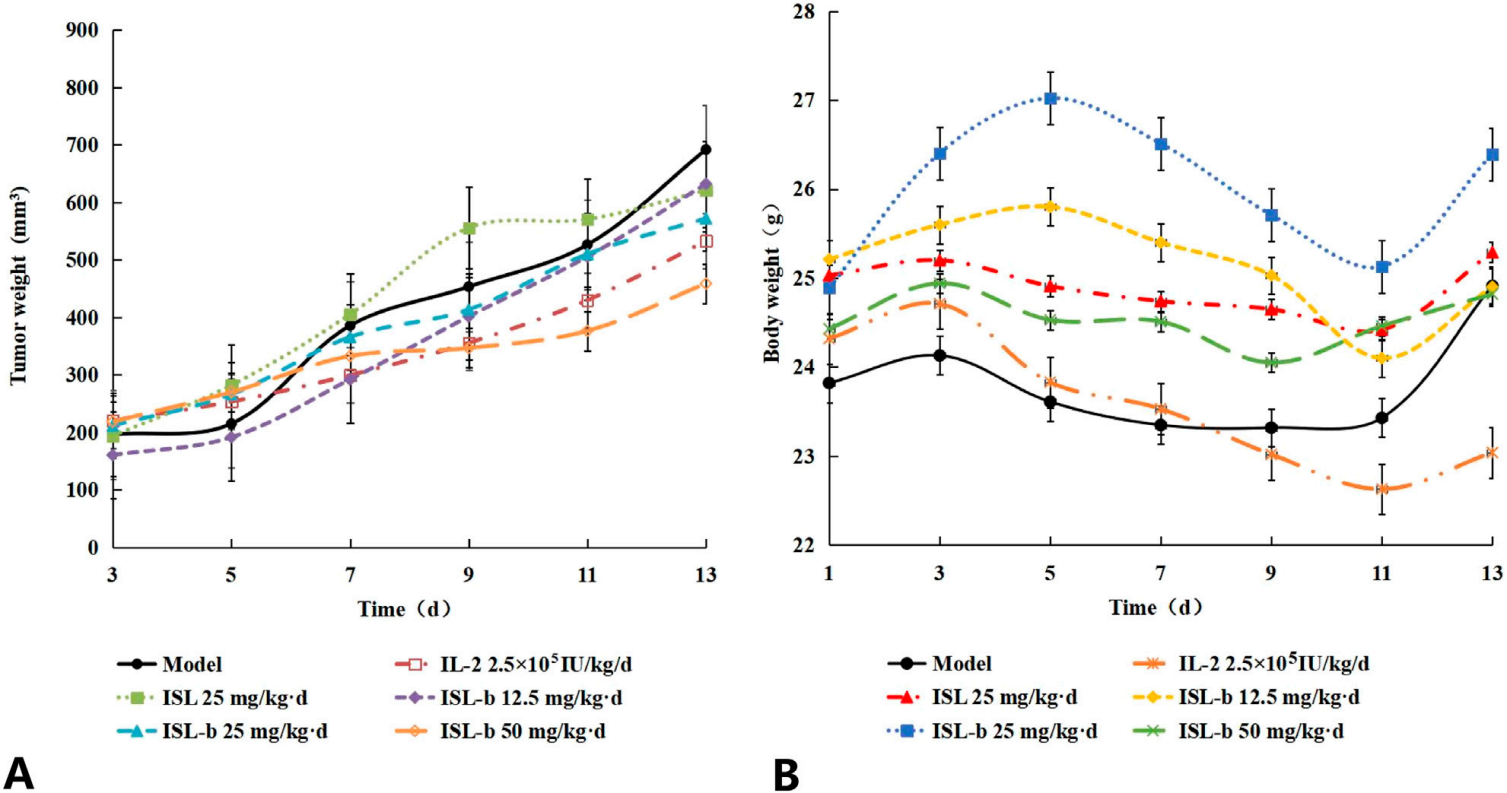
**(A)** Effect of isoglycosides and their derivatives on body weight changes in Caki-1 loaded mice, **(B)** Body weight changes in tumor-bearing mice (x ± s, n = 10).

ISL-b was able to inhibit the growth of tumor volume in Caki-1 loaded mice, both of which were significantly different (*P* < 0.01 or *P* < 0.05) compared with the model control group, with the smallest tumor volume of Caki-1 in the ISL-b 50 mg/kg-d dose group (Table 11) (Figure 8A).

**TABLE 11:** Changes in tumor volume of Caki-1 tumor-bearing mice (x ± s, n = 10, Unit: mm^2^).

Day	Model	IL-2 2.5 × 10^5^IU/kg/d	ISL 25 mg/kg-d	ISL-b		
				12.5 mg/kg-d	25 mg/kg-d	50 mg/kg-d
3	196.03 ± 11.02	220.23 ± 18.93*	193.42 ± 15.78	160.22 ± 12.04*	211.52 ± 16.04	218.44 ± 25.01*
5	214.9 ± 29.31	253.02 ± 21.27*	281.61 ± 18.33*	191.31 ± 18.13	265.65 ± 19.10*	269.61 ± 23.25*
7	385.27 ± 22.15	299.19 ± 25.82*	404.91 ± 25.16*	291.6 ± 26.07**	365.49 ± 23.16*	332.35 ± 28.79**
9	453.13 ± 24.07	355.36 ± 17.73**	555.60 ± 23.05**	401.22 ± 23.61*	413.39 ± 19.15*	346.37 ± 32.03**
11	526.28 ± 19.17	429.73 ± 22.16**	570.62 ± 26.13**	505.51 ± 20.15*	509.92 ± 22.47*	376.33 ± 31.02**
13	691.66 ± 30.36	532.78 ± 23.56**	620.70 ± 40.25**	631.98 ± 31.25**	572.14 ± 29.13**	458.53 ± 27.36**

Data are expressed as mean ± standard deviation (x ± s), n = 10.

Compared with the model group, *indicates P < 0.05 (significant difference), **indicates P < 0.01 (extremely significant difference).

Statistical method: One-way analysis of variance (ANOVA) with Bonferroni post-hoc test.

#### 4.7.2 Effect of isoglycosides and their derivatives on body weight changes in Caki-1 loaded mice

The model group of mice exhibited an increase in body weight over a duration of 13 days. In contrast, the IL-2 group experienced a decrease in body weight due to the drug’s toxic side effects, which resulted in reduced food intake. Conversely, the body weight of mice in the ISL-b dose groups remained stable. When compared to the model control group, no significant differences were observed in any of these groups (P > 0.05) (Table 12) (Figure 8B).

**TABLE 12:** Body weight changes in tumor-bearing mice (x ± s, n = 10, Unit: g).

Day	Model	IL-2 2.5 × 10^5^IU/kg/d	ISL 25 mg/kg-d	ISL-b		
				12.5 mg/kg-d	25 mg/kg-d	50 mg/kg-d
1	23.82 ± 0.95	24.32 ± 0.88	25.03 ± 1.12	25.21 ± 0.75	24.89 ± 0.48	24.43 ± 0.39
3	24.13 ± 1.02	24.71 ± 0.71	25.20 ± 1.03	25.60 ± 1.14	26.40 ± 0.74	24.94 ± 0.84
5	23.61 ± 0.87	23.83 ± 0.93	24.91 ± 0.89	25.80 ± 0.91	27.02 ± 0.82	24.53 ± 0.65
7	23.35 ± 0.76	23.53 ± 0.89	24.74 ± 0.77	25.40 ± 0.98	26.51 ± 0.68	24.51 ± 1.14
9	23.32 ± 0.85	23.02 ± 1.04	24.65 ± 0.94	25.03 ± 1.22	25.71 ± 0.53	24.05 ± 0.72
11	23.43 ± 1.01	22.63 ± 0.62	24.42 ± 0.86	24.10 ± 0.65	25.13 ± 0.71	24.46 ± 0.47
13	24.91 ± 0.91	23.04 ± 0.79	25.29 ± 0.92	24.90 ± 0.53	26.39 ± 1.17	24.82 ± 0.57

Data are expressed as mean ± standard deviation (x ± s), n = 10.

Compared with the model group, *indicates P < 0.05 (significant difference), **indicates P < 0.01 (extremely significant difference).

Statistical method: One-way analysis of variance (ANOVA) with Bonferroni post-hoc test.

#### 4.7.3 Effect of drugs on Caki-1 tumor tissue morphology

**FIGURE 9:**
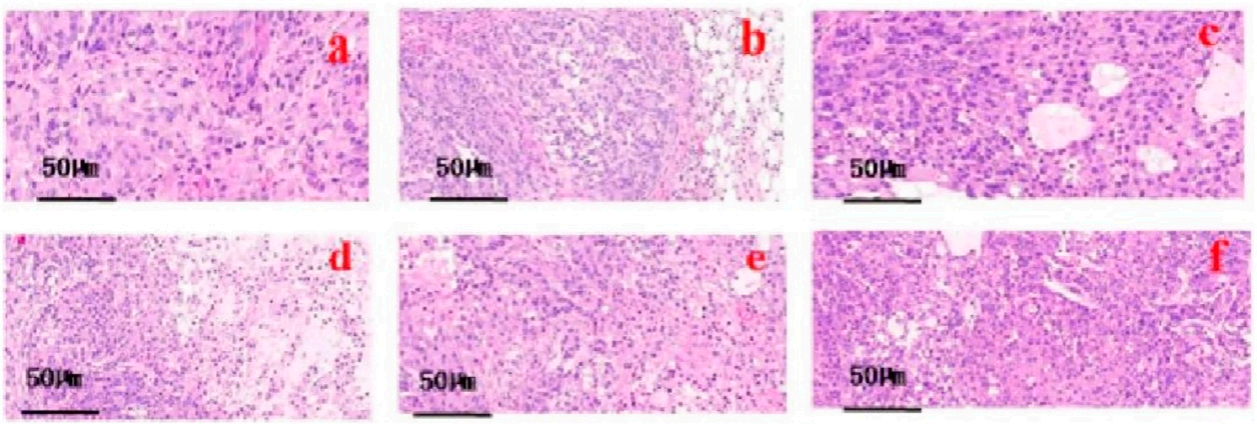
HE staining microscope of isoliquiritigenin and its derivatives on tumor tissue. **(a)** model group; **(b)** IL-2 group; **(c)** ISL group; **(d)** ISL-b high dose group; **(e)** ISL-b medium dose group; **(f)** ISL-b low dose group.

The histochemical results of hematoxylin and eosin (HE) staining of tumor tissue,it reveals that in the model group, cancer cells exhibit vigorous growth. The tumor cells are characterized by polygonal or spindle shapes, with pronounced nuclear polymorphism, displaying round, elliptical, or irregular shapes. The nuclear schizogenic image is evident, indicating a high nuclear-to-cytoplasmic ratio, while the cytoplasm appears light iridescent red. Tumor cells are distributed in patches and clusters, arranged closely together. In the IL-2 group and the ISL, ISL-b groups, an increase in apoptotic cells is observed, with these cells appearing separated from neighboring cells, and their nuclei are either condensed or even absent. In the ISL-b low and middle dose groups, tumor cells exhibit mild degenerative changes, with occasional large areas of necrosis. Conversely, tumor cells in the ISL-b high dose group display poorly defined contours, reduced density, and an increased necrotic area (Figure 9).

#### 4.8 The effect of the drug on the expression of proteins related to the NF-κB signaling pathway

**FIGURE 10:**
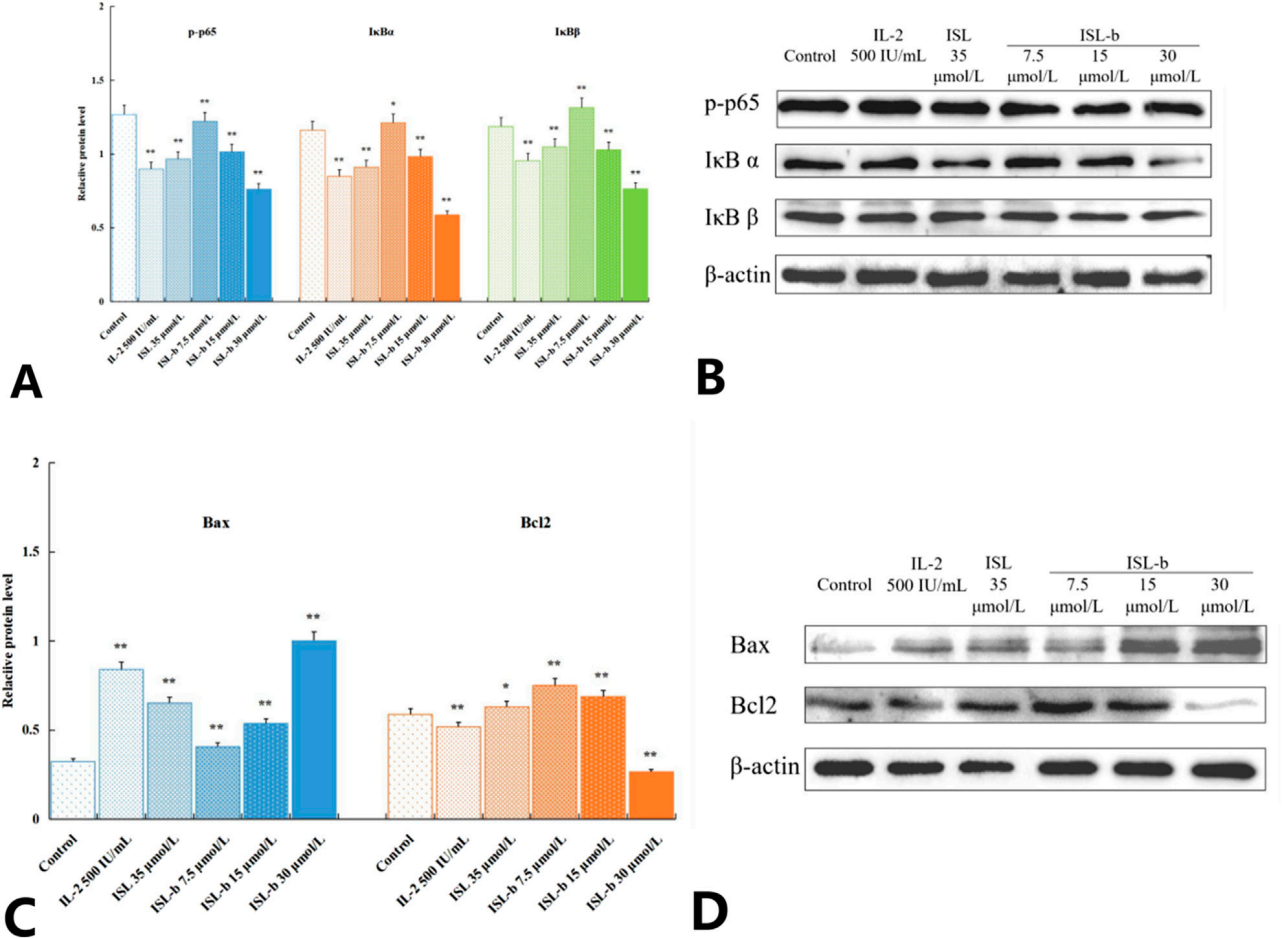
Effect ofp-p65, IκBα, IκBβ protein expression ratio of Caki-1 cells (*x* ± *s*, *n* = 3) **P < 0.01 Compared with control **(A,B)**. Effect of Bax, Bcl-2protein expression ratio of Caki-1 cells (*x* ± *s*, *n* = 3) **P < 0.01 Compared with control **(C,D)**.

Compared with the blank control group, after treatment with different concentrations of IL-2, ISL and ISL-b for 24 h, the expression levels of p-p65, IκBα and IκBβ proteins in Caki-1 cells decreased. Among them, after ISL-b treatment, the expression levels of p-p65, IκBα and IκBβ proteins showed a negative correlation with concentration; the expression level of Bax, a pro-apoptotic protein downstream of the NF-κB signaling pathway, increased and showed a positive correlation with concentration; the expression of anti-apoptotic protein Bcl-2 decreased and showed a negative correlation with concentration. Statistical tests showed that there were statistically significant differences between the experimental groups and the blank control group (P < 0.05). The Bcl-2/Bax protein expression ratio was 1.8406 in the blank control group and 0.2654 in the high-dose ISL-b group (Figures 10A–D).

### 4.9 Differential analysis of intestinal flora in tumor-bearing mice

#### 4.9.1 Alpha-diversity analysis

There was no significant difference in the number of observed species between the model group and the IL-2 drug group. However, the model group exhibited higher Shannon diversity, greater community diversity, and a more even species distribution. When comparing the model group to the ISL-b high-dose group, the number of observed species was greater in the ISL-b high-dose group, indicating a higher species richness in this group. In contrast, the model group showed a lower number of observed species when compared to the ISL-b medium and low-dose groups, suggesting a reduced species richness in these latter groups. Furthermore, the Chao1 index indicated that the total number of species present in the community samples was significantly greater in the ISL-b high-dose group (Table 13).

**TABLE 13:** Comparison of alpha diversity between the model group and each dosing group.

Sample name	Observed _species	Shannon	Simpson	chao1	ACE	Goods_coverage	Tree
Model group	497	5.038	0.930	542.493	542.354	0.999	56.845
IL-2	439	4.285	0.905	490.183	528.271	0.998	52.712
ISL-b low dose	387	4.373	0.908	418.544	427.056	0.999	45.379
ISL-b low dose	379	4.158	0.881	411.051	419.571	0.999	41.396
ISL-b Medium Dose	556	5.010	0.936	592.404	601.399	0.999	55.471
ISL-b medium dose	454	4.474	0.91	479.536	493.317	0.999	53.957
ISL-b High dose	640	4.974	0.922	698.579	721.151	0.998	85.293
ISL-b High dose	752	4.720	0.897	852.837	854.749	0.997	84.491
ISL	478	4.717	0.895	544.444	544.591	0.998	55.499
ISL	412	4.770	0.925	446.424	464.821	0.999	48.595

*P < 0.05 Compared with model; **P < 0.01 Compared with model.

#### 4.9.2 BetaDiversity analysis (BetaDiversity)

**FIGURE 11:**
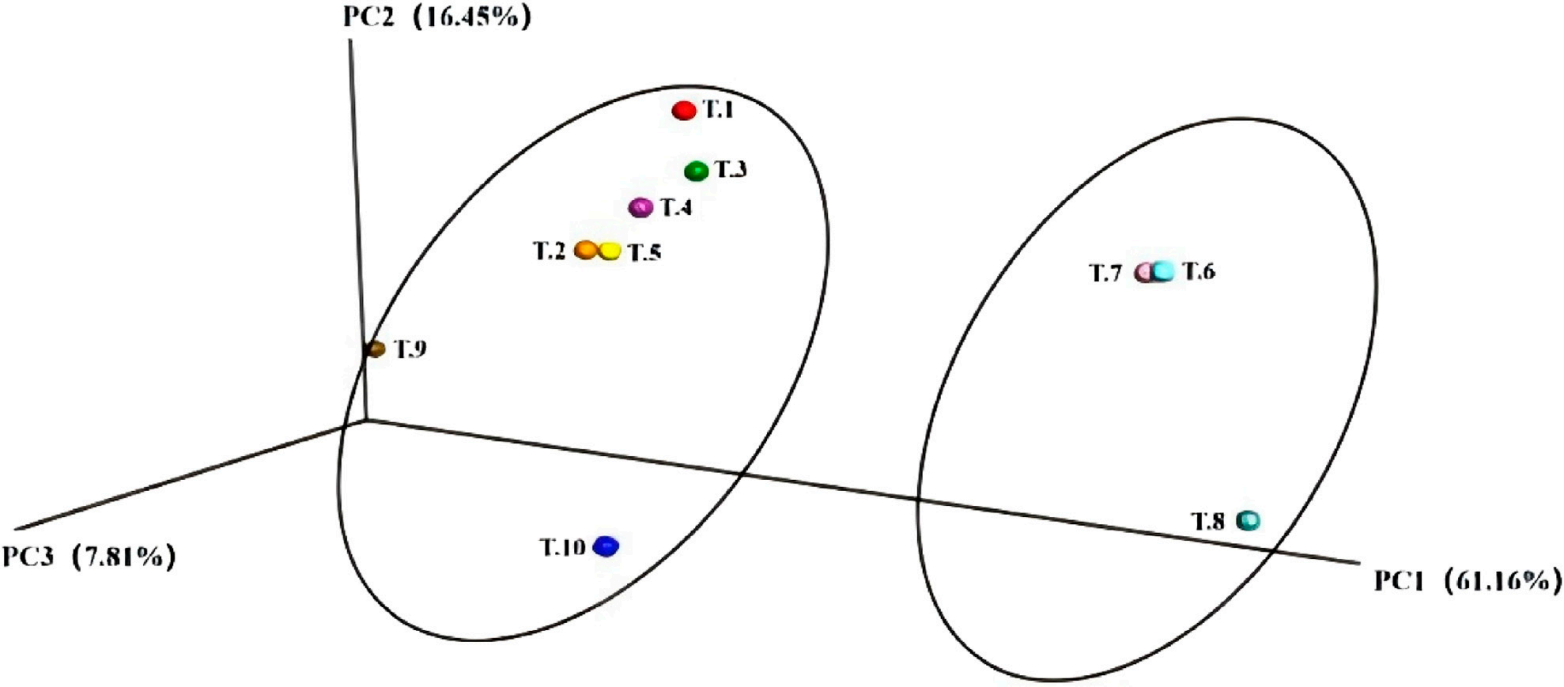
PCoA Analysis PcoA. (Note: T1: model group; T2: IL-2 group; T3: ISL-b low-dose group; T4: ISL-b low-dose group; T5: ISL-b medium-dose group; T6: ISL-b medium-dose group; T7: ISL-b high-dose group; T8: ISL-b high-dose group; T9: ISL group; T10: ISL group).

The model group, IL-2 group, ISL, and low-dose ISL-b group can be clustered together, while the medium and high-dose ISL-b groups form a separate cluster. This observation indicates significant differences in species composition within the intestinal feces. Furthermore, the high-dose ISL-b group is positioned further from the model group, suggesting that the species composition between the high-dose ISL-b group and the model group exhibits greater differentiation (Figure 11).

#### 4.9.3 Analysis of the structural composition of the intestinal flora in mice

**FIGURE 12:**
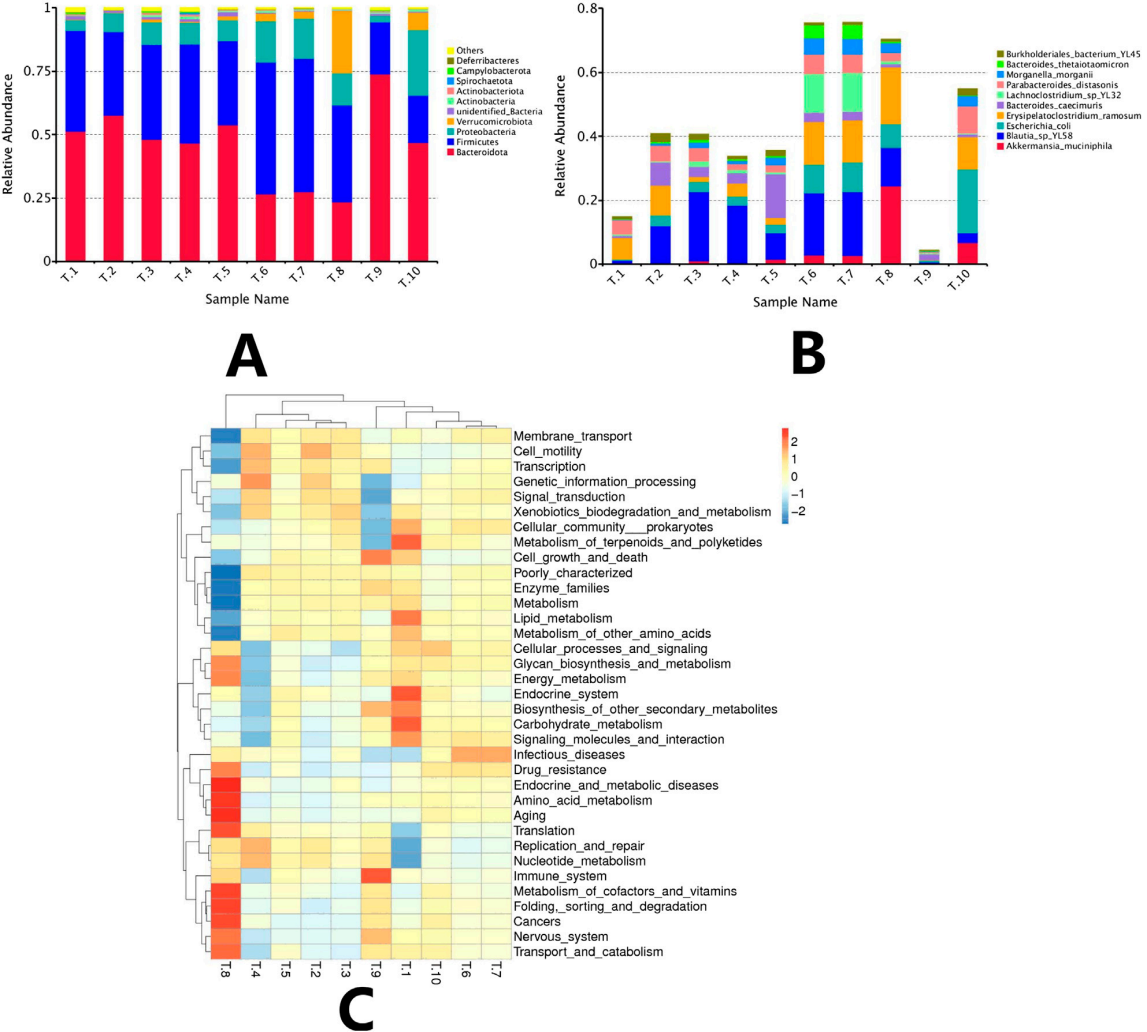
A Composition of abundance at the phylum level of the bacterial community **(A)**. Species-level abundance composition of the bacterial population **(B)**. (Note: T1: model group; T2: IL-2 group; T3: ISL-b low-dose group; T4: ISL-b low-dose group; T5: ISL-b medium-dose group; T6: ISL-b medium-dose group; T7: ISL-b high-dose group; T8: ISL-b high-dose group; T9: ISL group; T10: ISL group). KEGG functional enrichment of differential genes in intestinal flora **(C)**. (Note: T1: model group; T2: IL-2 group; T3: ISL-b low-dose group; T4: ISL-b low-dose group; T5: ISL-b medium-dose group; T6: ISL-b medium-dose group; T7: ISL-b high-dose group; T8: ISL-b high-dose group; T9: ISL group; T10: ISL group).

Based on the results of species annotation, abundance histograms were plotted, and the top 10 microbial taxa at both the phylum and species levels were statistically analyzed in the model group and each drug group, respectively. The results indicated that the dominant phyla in each group were Bacteroidetes and Firmicutes. The model group exhibited the highest proportions of Bacteroidetes and Firmicutes, which together accounted for 90.9522%. In contrast, Verrucomicrobiota accounted for 0.1771% in the model group; however, its abundance increased in the drug groups with rising drug concentrations. Notably, the ISL-b high-dose group demonstrated the highest percentage of Verrucomicrobiota at 24.49%. According to the species abundance histograms, there was a significant increase in the abundance of Akkermansia muciniphila and Blautia sp. YL58 at the species level, with A. muciniphila showing a corresponding increase in abundance with higher drug concentrations. In the high-dose ISL-b group, A. muciniphila accounted for 24.48%, which was 204 times greater than that observed in the model group (Figures 12A–C).

#### 4.9.4 Analysis of the functional composition of the intestinal flora in each group of mice

KEGG functional enrichment analysis revealed that the differential genes of gut flora in the high-dose ISL-b drug group were primarily enriched in pathways related to cancers, translation, replication and repair, and nucleotide metabolism. In contrast, the model group exhibited enrichment in pathways associated with infectious diseases, drug resistance, endocrine and metabolic diseases, amino acid metabolism, and aging.

## 5 Discussion

China boasts extremely rich licorice resources with dual medicinal and ecological values, making the efficient development of its medicinal components (e.g., isoliquiritigenin, ISL) a critical research focus for both resource utilization and pharmaceutical innovation.

First, in optimizing ISL extraction from licorice, this study employed *Aspergillus niger* solid-state fermentation, achieving an extraction rate nearly 9-fold higher than traditional methods—a result that resolves a long-standing limitation of conventional techniques. As (Yu et al., 2020) documented, ISL in licorice exists in both glycogen-conjugated bound and free forms, but traditional methods only isolate free ISL, leading to underutilization of licorice resources. In contrast, our findings clarify that *A. niger* secretes β-glucosidase during metabolism: this cellulase first disrupts licorice cell walls to release intracellular ISL (Pattanaik et al., 2022) and then hydrolyzes β-1,4 glycosidic bonds to convert bound ISL into free ISL (Cheng and DeYonker, 2022; Wu W. et al., 2022). While (Yang et al., 2022) previously reported that microbial enzymes can transform bound active components, our work advances this understanding by specifying the exact enzyme (β-glucosidase) and its target substrate (β-1,4 glycosidic bonds in licorice ISL)—filling a gap in the “microbe-enzyme-licorice ISL” interaction pathway that prior studies overlooked. Critically, few existing studies have quantified β-glucosidase’s efficacy in licorice ISL extraction; our 9-fold yield increase provides concrete quantitative evidence, confirming the enzyme’s role is not a general “efficiency boost” but a targeted solution to bound ISL underutilization.

Second, the chemical modification of ISL to synthesize ISL-b addresses key limitations of natural ISL and advances antitumor drug development. ISL, a hydroxychalcone with a C_15_H_12_O_4_ molecular formula (Sun et al., 2021) and a flexible two-benzene-ring structure (Zhang et al., 2018), suffers from low oral absorption (Wang et al., 2018), photothermal/acidic instability, and non-specific action—barriers to clinical application. Our strategy of introducing methoxyl groups (-OCH_3_) at the C4 and C4′ positions resolves these issues: the lone pair of electrons on methoxyl oxygen forms p-π conjugation with the benzene ring, enhancing molecular electron cloud density and potentially strengthening binding affinity with the p65 protein in the NF-κB signaling pathway. Experimental results validate this modification: the ISL-b high-dose group exhibited a 56.3% tumor inhibition rate in Caki-1 tumor-bearing mice, with HE staining showing increased tumor cell apoptosis/necrosis, and routine blood tests/liver/kidney histology confirming no toxic side effects. Western blot analysis further supported the mechanism: ISL-b downregulated p-p65, IκBα, and IκBβ (NF-κB pathway proteins) in a concentration-dependent manner, upregulated the pro-apoptotic protein Bax, and downregulated the anti-apoptotic protein Bcl-2. However, critical gaps remain: derivative synthesis conditions were not systematically optimized, and exploration of other functional groups (e.g., ethoxy substitutions) is limited. Future work must address these to fully establish ISL’s structure-activity relationship (SAR), a step essential for translating ISL derivatives into clinical use.

Third, ISL-b’s regulation of intestinal flora reveals a dual antitumor mechanism, enriching understanding of natural products’ antitumor action. A substantial body of research confirms bidirectional interactions between tumors and intestinal flora: the tumor microenvironment shapes flora composition (Sánchez-Alcoholado et al., 2021; Wu N. et al., 2022), while flora metabolites regulate tumor proliferation and immune evasion (Zhang et al., 2021; Liu et al., 2022), forming a pathological interaction network (Dusek et al., 2020). Our results align with this framework: the ISL-b high-dose group showed higher intestinal flora biodiversity (Chao1 index) than the model group, with increased abundance of *Micrococcus* wartii (phylum level) and Akkermansia muciniphila (species level). A. muciniphila—a mucin-degrading anaerobe (Kim et al., 2021; Yan J. et al., 2021) that produces propionic acid (Shin et al., 2021; Segura Munoz et al., 2022) —interacts with host intestinal tissues via Gpr43 to modulate immunity (Giannoudaki et al., 2019; Stražar et al., 2021). However, our study did not link propionic acid to specific host immune factors, a gap that future research should fill to fully unravel the “flora-metabolite-immunity-tumor” regulatory axis.

Synthesizing these findings, this study makes three key contributions: it provides a scalable, high-efficiency ISL extraction process using *A. niger* fermentation, addressing licorice resource underutilization; it validates ISL-b as a safe, potent antitumor derivative targeting the NF-κB pathway, laying a foundation for clear cell renal cell carcinoma treatment; and it uncovers ISL-b’s dual antitumor mechanism (direct pathway inhibition + indirect flora regulation), expanding insights into natural product pharmacology. These results not only advance basic research but also offer practical support for high-value licorice processing, aligning with the goal of sustainable medicinal resource development.

## 6 Conclusion

The process of extracting isoliquiritigenin from licorice was optimized using solid-state fermentation with Aspergillus niger and response surface methodology, resulting in a straightforward extraction process with a high yield. The results showed that the optimal extraction conditions were a pH of 3.7, a solid-liquid ratio of 1:2, an inoculation concentration of Aspergillus niger of 1.5 × 10^6^. After 4 days of fermentation, the yield of isoliquiritigenin was 1.53 mg/g. Isoliquiritigenin served as the substrate for structural modification, wherein the hydroxyl groups at both the C4 and C4′ positions underwent the anticipated acylation or methylation reactions. ISL-b exhibited a growth inhibitory effect on Caki-1 cells derived from renal clear cell carcinoma, which intensified with increasing dosage. Furthermore, ISL-b effectively inhibited tumor growth in Caki-1 homozygous mice, reduced tumor volume, and preserved the body weight of the mice, indicating good drug safety. Variations in ISL-b concentration significantly influenced protein expression levels. Specifically, as ISL-b concentration increased, the expression levels of p-p65, IκBα, and IκBβ proteins decreased, demonstrating a negative correlation. In contrast, the expression level of the pro-apoptotic protein Bax, which is downstream of the NF-κB signaling pathway, increased, showing a positive correlation with ISL-b concentration. Additionally, the expression level of the anti-apoptotic protein Bcl-2 exhibited a negative correlation with ISL-b concentration. Mice in the high-dose ISL-b group displayed significant alterations in intestinal flora, with notable upregulation of mucinophilic Akkermansia muciniphila and Blautia sp. YL58. Furthermore, significant changes in the functional profiles of differential genes within the intestinal flora were observed in the high-dose ISL-b group.

Based on the results and limitations of this study, future research can be deepened in the following unexplored directions: First, in terms of the fermentation process, although core parameters such as pH and solid-liquid ratio have been determined, the synergistic effects of fermentation temperature (30 °C ± 2 °C) with other parameters on the activity of Aspergillus niger β-glucosidase and the accumulation of isoliquiritigenin (ISL) have not been systematically explored. Moreover, the current laboratory scale (100 mL Erlenmeyer flasks) does not involve issues such as uneven mass and heat transfer and solvent recovery in pilot-scale and industrial scale-up. In subsequent studies, it is necessary to supplement temperature gradient experiments and establish a 50-L scale fermentation system to verify the stability of the optimized process. Second, in the aspect of pharmacological research on ISL derivatives, it is known that ISL-b exerts antitumor effects by inhibiting the NF-κB signaling pathway, but it has not been verified whether ISL-b activates the ROS-mediated oxidative damage pathway (e.g., detecting ROS levels and caspase-3/9 expression in Caki-1 cells). Additionally, there is a lack of pharmacokinetic parameters (half-life, bioavailability) and long-term toxicity data (effects of 3-month repeated administration on hepatic and renal function). Relevant experiments need to be improved to support clinical translation. Third, in terms of derivative structure and intestinal flora mechanism, the structural characterization of ISL-a/-b/-c lacks high-resolution mass spectrometry (HRMS) data and complete 13C-NMR carbon skeleton assignment, and the structure-activity relationship (SAR) of other substituents (e.g., ethoxy groups) has not been explored. Meanwhile, regarding the mechanism by which ISL-b upregulates the abundance of Akkermansia muciniphila, the interaction between the metabolites of this bacterium (e.g., propionic acid) and host immune factors (e.g., IL-22) has not been linked. In subsequent studies, it is necessary to supplement structural confirmation experiments and explore the “flora-metabolite-immunity-tumor” regulatory network.

## Data Availability

The original contributions presented in the study are publicly available. This data can be found here: https://www.ncbi.nlm.nih.gov/sra/PRJNA1354458.
