# Supplementary figures and images for: Optimization of response surface methodology for the extraction of isoliquiritigenin from Aspergillus niger solid-state fermentation of licorice and its antitumor effects
Source: Front Pharmacol. 2025 Nov 11;16:1629167. doi: 10.3389/fphar.2025.1629167 (PMC12644103; doi:10.3389/fphar.2025.1629167)

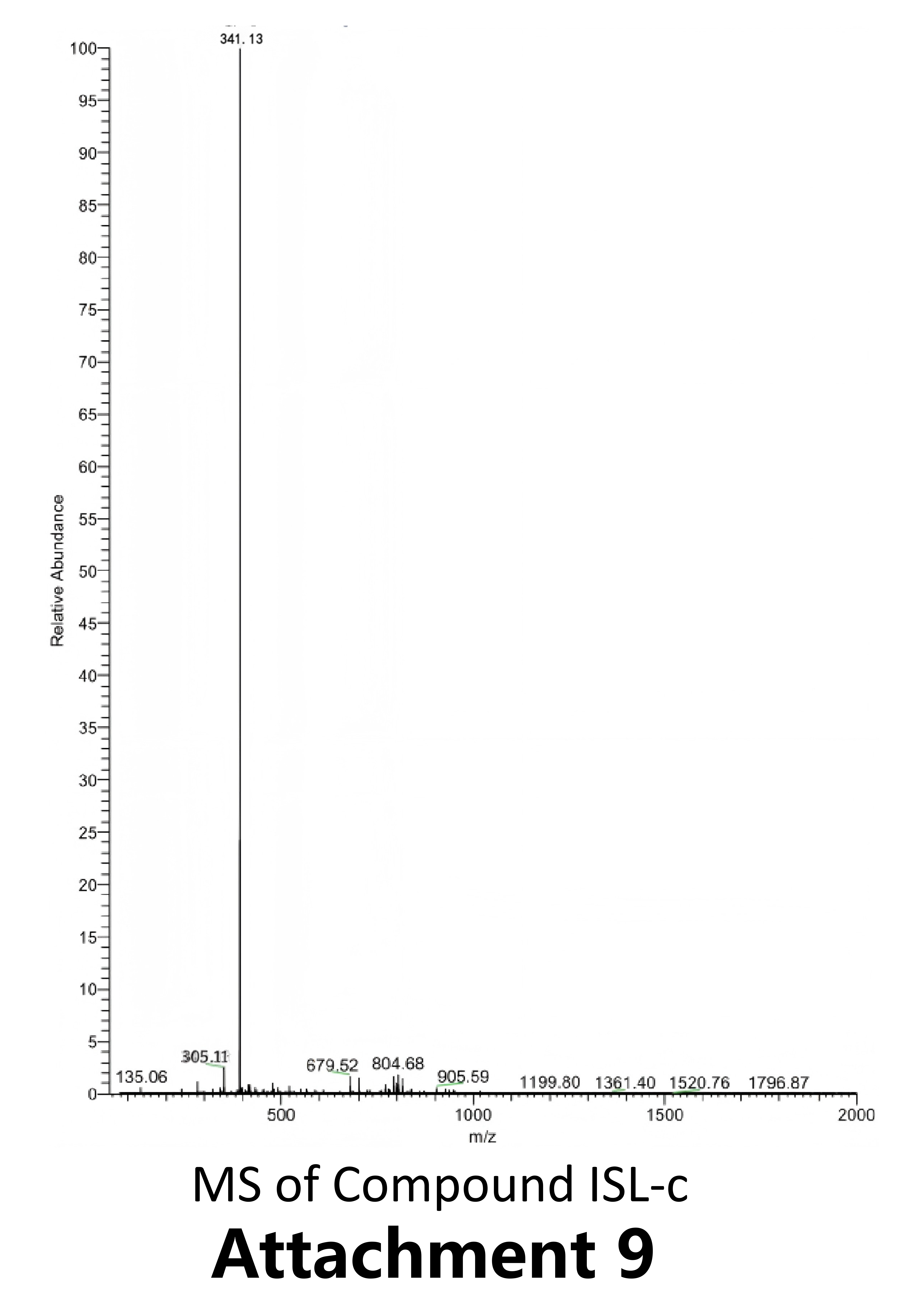

Supplement: Supplementary file 3 [file Image9.tiff]

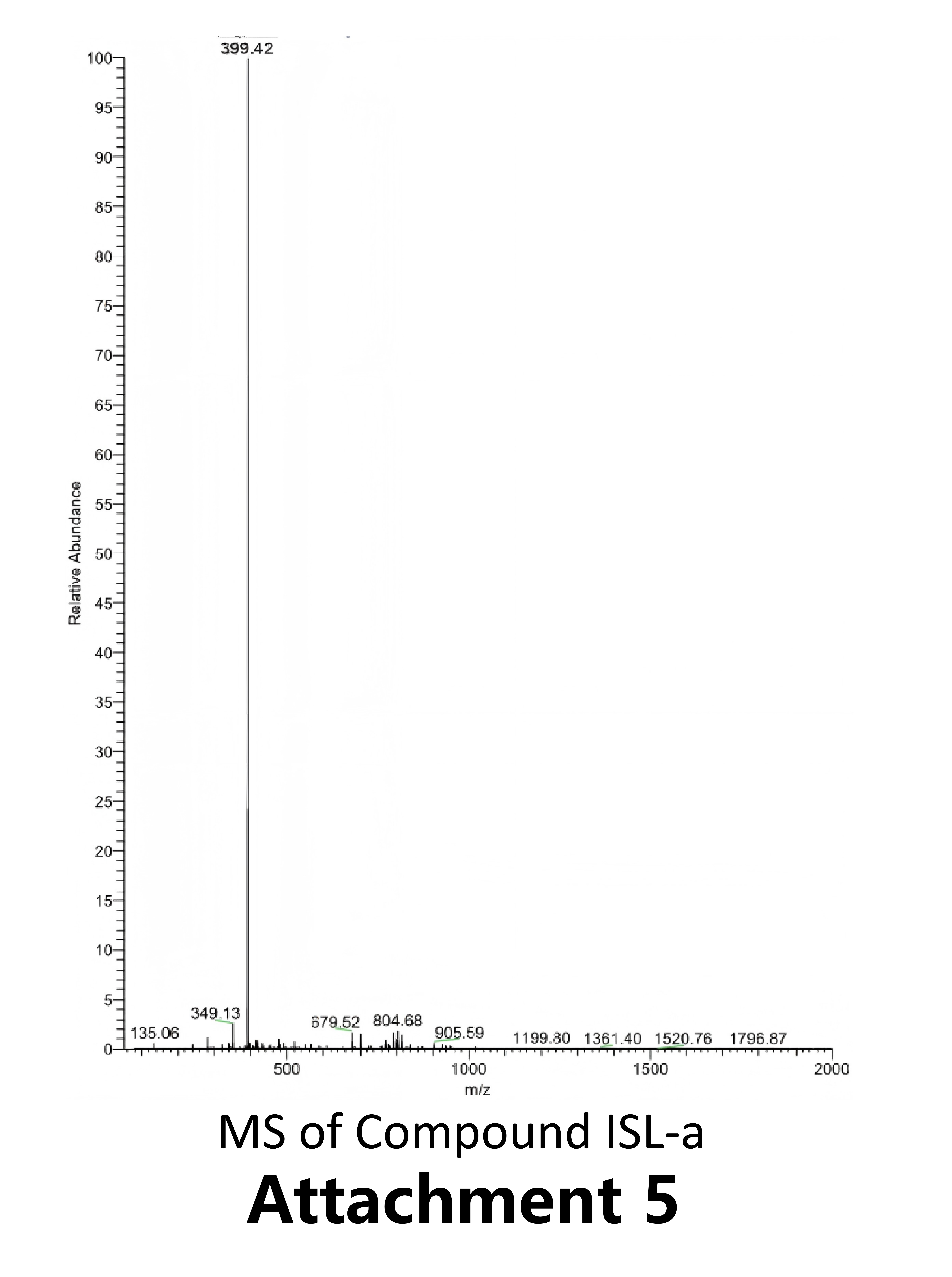

Supplement: Supplementary file 5 [file Image5.tiff]

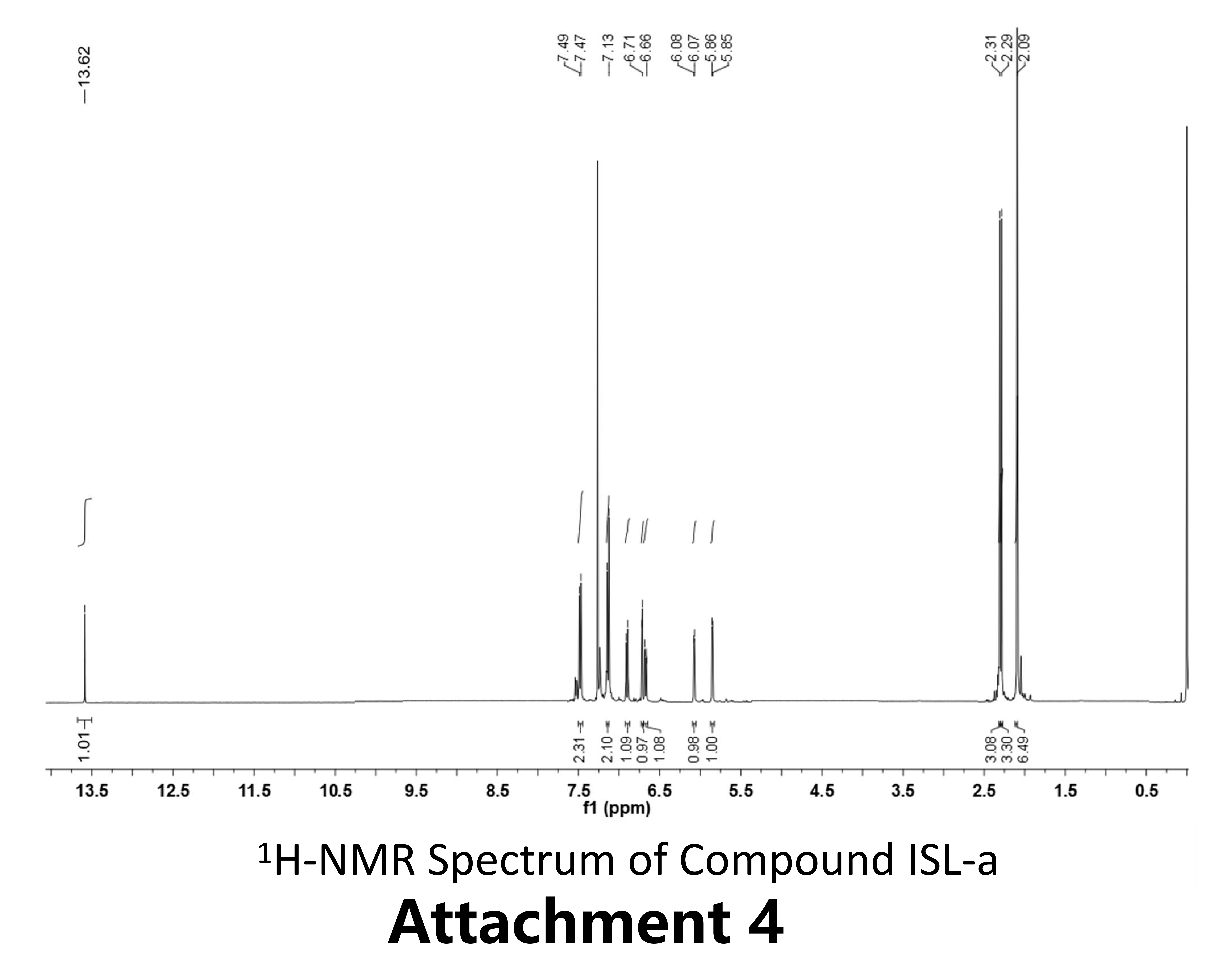

Supplement: Supplementary file 9 [file Image4.tiff]

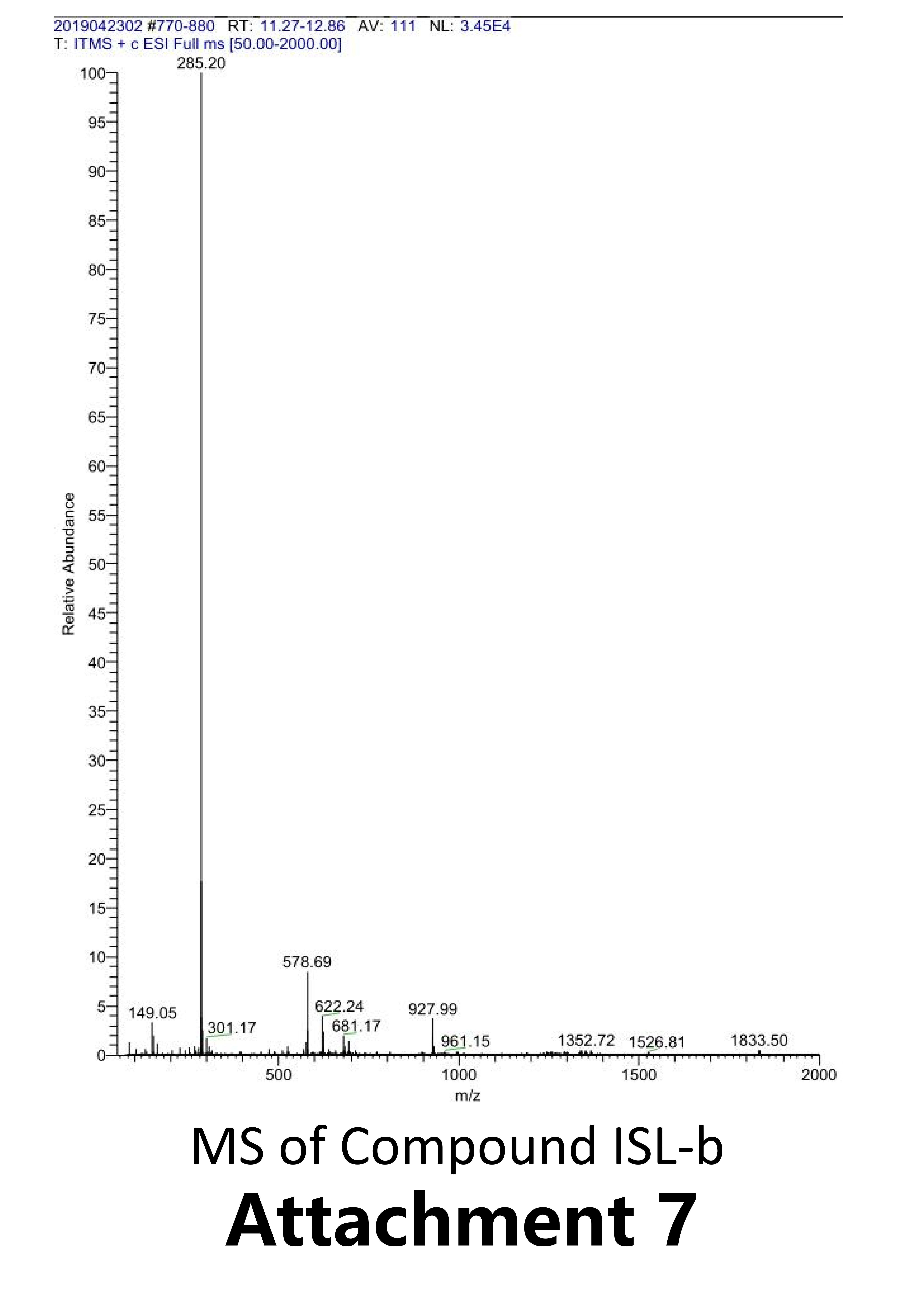

Supplement: Supplementary file 10 [file Image7.tiff]
