# Supplementary material for: Optimization of response surface methodology for the extraction of isoliquiritigenin from Aspergillus niger solid-state fermentation of licorice and its antitumor effects
Source: Front Pharmacol. 2025 Nov 11;16:1629167. doi: 10.3389/fphar.2025.1629167 (PMC12644103; doi:10.3389/fphar.2025.1629167)
Supplement: Supplementary file 4 [file Table1.docx]

**Supplementary Tab. 1A 1D and 2D Data of isoliquiritigenin (DMSO-d6, Jin Hz)**

| **No.** | **δH** | **δc** | **COSY** | **HMBC** | **Coupling Relationship Notes** |
| --- | --- | --- | --- | --- | --- |
| 1 |  | 112.88 |  |  | No hydrogen signal (A ring C1 is quaternary carbon), no coupling analysis required |
| 2 |  | 165.68 |  |  | No hydrogen signal (A ring C2 is quaternary carbon, connected to hydroxyl group), no coupling analysis required |
| 3 | 6.28 (d, J = 2.3 Hz, 1H) | 102.47 | H-5 | C1, C2, C4, C5 | Corresponding to A-ring C3-H: Only exhibits meta coupling with C5-H (J=2.3 Hz, typical meta coupling constant range 2-3 Hz), no ortho hydrogen coupling. Directly confirms the 1,3,5-substituted “2,4-dihydroxy” configuration of the A-ring. - trisubstituted benzene ring characteristic (C2 and C4 are quaternary carbons, only C3 and C5 contain hydrogen) |
| 4 | 6.41 (dd, J= 8.9, 2.3 Hz. | 164.85 |  |  | No hydrogen signal (A ring C4 is a quaternary carbon, connected to a hydroxyl group), no coupling analysis required |
| 5 | 1H) | 107.98 | H-3, H-6 | C1, C3 | Corresponding to A-ring C5-H: exhibits ortho coupling with C6-H (J=8.9 Hz, typical ortho coupling range 6-10 Hz) and meta coupling with C3-H (J=2.3 Hz). This dual coupling pattern further validates the A-ring's 1,3,5-trimethylbenzene structure with 2,4-dihydroxy substitution, ruling out alternative substitution patterns. - dihydroxy substitution" in the 1,3,5-trimethyl structure, excluding other substitution patterns. |
| 6 | 8.17 (d, J = 9.0 Hz, 1H) | 132.76 | H-5 | C1, C2, C3, C7. | Corresponding to A-ring C6-H: Exhibits only ortho coupling with C5-H (J=9.0 Hz), with no other coupling signals; combined with HMBC coupling to C7 carbonyl carbon, this confirms C6 is adjacent to the carbonyl group (electron effect causing δH increase), supporting the direct connection between “A-ring - C7 carbonyl”. |
| 7 | 7.75 to 7.71 (d , J= 15.2 Hz. | 191.43 |  |  | No hydrogen signal (C7 is the carbonyl carbon), eliminating the need for coupling analysis |
| 8 | 4H)  7.80 to 7.76 (d , J= 15.2 Hz. | 117.30 | H-9 | C7, C9, C1' | Corresponding to side chain C8-H: Only exhibits trans double bond coupling with C9-H (J=15.2 Hz, typical range for trans double bond coupling constant is 14-16 Hz), confirming the double bond is in the E configuration; Combined with HMBC coupling to B-ring C1', confirms direct linkage “C8 double bond - B-ring C1'”, supporting the chalcone “ring - carbonyl - double bond - ring” core structure |
| 9 | 4H) | 144.17 | H-8 | C7, C8, C1', C2'. | Corresponding side chain C9-H: Exhibits only trans coupling with C8-H (J=15.2 Hz), consistent with C8-H coupling constant, further validating the trans configuration of the double bond; HMBC coupling with B-ring C2' confirms stable linkage between the B-ring and the double bond |
| 1' |  | 125.64 |  |  | No hydrogen signal (B-ring C1' is quaternary carbon), eliminating need for coupling analysis |
| 2' | 6.84 (d , J= 8.6 Hz, 1H) | 131.13 | H-3' | C1', C3', C4' | Corresponding B-ring C2'-H: Exhibits ortho coupling with C3'-H (J=8.6 Hz), consistent with the para-substituted benzene ring characteristic of B-ring “4'-hydroxy substitution” (C2', C6' are symmetric hydrogens, C3' and C5' are symmetric hydrogen atoms), no other coupling signals present, ruling out interference from multiple substituents on the B ring |
| 3' | 7.76 (d , J= 8.6 Hz, 1H) | 115.73 | H-2' | C1', C2', C4' | Corresponding to B ring C3'-H: exhibits ortho coupling with C2'-H (J=8.6 Hz), coupling constant consistent with C2'-H, validating the para-substituted pattern on the B ring; Combined with HMBC and C4'-quaternary carbon coupling, this confirms hydroxyl substitution at the C4' position of ring B. |
| 4' |  | 160.16 |  |  | No hydrogen signal (ring B C4' is a quaternary carbon bearing a hydroxyl group), eliminating the need for coupling analysis. |
| 5' | 7.76 (d , J= 8.6 Hz, 1H) | 115.73 | H-6' | C1', C6', C4' | Corresponding to B-ring C5'-H: Exhibits ortho coupling with C6'-H (J=8.6 Hz), symmetrical with C3'-H signal, further confirming B-ring para substitution structure. No additional coupling proves only C4' position bears hydroxyl on B-ring. |
| 6' | 6.84 (d , J= 8.6 Hz, 1H) | 131.13 | H-5' | C1', C5', C4' | Corresponding to B-ring C6'-H: Exhibits an ortho coupling with C5'-H (J=8.6 Hz), symmetrical with the C2'-H signal. This fully validates the para-substituted “4'-hydroxy” structure on the B-ring, with no interference from other substituents. |
| 2-OH | 13.61 (s, 1H) |  |  | C1, C2, C3, C7. | Corresponding to A-ring C2-OH: Single peak without coupling (s peak). Intramolecular hydrogen bonding with C7 carbonyl (O-H…O=C) restricts proton exchange, resulting in a sharp peak shape. Significantly elevated δH (13.61 ppm) confirms this as the characteristic signal for the C2 hydroxyl on the A-ring, ruling out modification at the C2 position. |
| 4-OH | 10.68 (s, 1H) |  |  |  | Corresponding to A-ring C4-OH: Single peak without coupling (s-peak), no intramolecular hydrogen bonding (due to distance from C7 carbonyl), protons readily exchange with solvent causing slightly broadened peak shape; δH within typical phenolic hydroxyl range (10–11 ppm), confirming the presence of the unmodified A-ring C4 hydroxyl group |
| 4'-OH | 10. 14 (s, 1H) |  |  |  | Corresponding to B-ring C4'-OH: Single peak without coupling (s-peak), no intramolecular hydrogen bonding, broad peak due to easy proton exchange; δH close to A-ring C4-OH, corroborating the pre-modified state of the B-ring C4'-OH hydroxyl group, consistent with the chemical environment of a para-substituted hydroxyl on the B-ring. |

**Supplementary Tab.1B Proton / Carbon Chemical Shift and Environmental Assignment Table**

| **Compound** | **ID** | **Proton/Carbon Type** | **δH**  **(ppm)[Peak Shape，J(Hz)]** | **δC (ppm)** | **Proton/Carbon Environment Description** | **Assignment Basis** |
| --- | --- | --- | --- | --- | --- | --- |
| ISL | 2-OH | Hydroxyl proton | 13.61 (s, 1H) | - | A ring C2 hydroxyl forms an intramolecular hydrogen bond with C7 carbonyl | The peak shape is sharp (hydrogen bonding restricts proton exchange), with δ values significantly higher than other hydroxyl groups (typical phenolic hydroxyl δ 10.0–11.0 ppm); HMBC shows no carbon coupling (no corresponding carbon signal for the hydroxyl group), consistent with intramolecular hydrogen bonding characteristics. |
|  | H-3 | A-ring proton | 6.28 (d, J=2.3, 1H) | 102.47 | A ring C3 proton exhibits only meta coupling with C5 proton | COSY coupling only with H-5 (J=2.3 Hz, meta coupling constant), no ortho proton coupling; HMBC coupling with C1/C2/C4/C5 (adjacent quaternary carbons on A-ring), consistent with the “2,4-dihydroxy” substitution pattern on the A-ring |
|  | H-5 | A-ring proton | 6.41 (dd, J=8.9, 2.3, 1H) | 107.98 | A ring C5 proton exhibits ortho coupling with C6-H and meta coupling with C3-H | COSY shows coupling with H-3 (J=2.3 Hz) and H-6 (J=8.9 Hz, ortho coupling constant); HMBC coupling with C1/C3 confirms the chemical environment at the C5 position of the A ring |
|  | H-6 | A-ring proton | 8.17 (d, J=9.0, 1H) | 132.76 | A ring C6 proton, ortho-coupled with C5-H, influenced by C7 carbonyl's electron-withdrawing effect | COSY shows coupling only with H-5 (J=9.0 Hz); δ value is elevated (typically δ 6.0–8.0 ppm for aromatic protons) due to deshielding from proximity to the C7 carbonyl (electron-withdrawing); HMBC coupling with C1/C2/C3/C7 confirms spatial proximity to the carbonyl |
|  | 4-OH | Hydroxyl proton | 10.68 (s, 1H) | - | A ring C4 hydroxyl group, no intramolecular hydrogen bond | Broad peak shape (proton prone to solvent exchange), δ value within typical range for phenolic hydroxyl; No coupling with 2-OH, confirming independent existence of A-ring hydroxyl |
|  | H-8 | Side-chain double bond proton | 7.76 (d, J=15.2, 1H) | 117.30 | Side chain C8 double bond proton, trans configuration (E-configuration) | COSY coupling with H-9 (J=15.2 Hz, characteristic coupling constant for trans double bonds); δ value lower than H-9 due to proximity to C7 carbonyl (electron-withdrawing); HMBC coupling with C7/C9/C1' confirms conjugation between the double bond and rings A and B |
|  | H-9 | Side-chain double bond proton | 7.80 (d, J=15.2, 1H) | 144.17 | Side chain C9 double bond proton, trans configuration, conjugated with B ring | COSY coupling with H-8 (J=15.2 Hz); higher δ value due to electron-donating effect from conjugation with ring B, increasing electron density; HMBC coupling with C7/C8/C1'/C2' verifies double bond linkage to ring B |
|  | H-2'/H-6' | B-ring proton | 6.84 (d, J=8.6, 2H) | 131.13 | B ring C2'/C6 proton, ortho-coupled C3'/C5'-H | COSY coupling with H-3'/H-5' (J=8.6 Hz, ortho coupling constant); δ value consistent with ortho-substituted benzene ring “ortho proton” characteristic; absence of other coupling signals confirms B ring as monosubstituted ortho |
|  | H-3'/H-5' | B-ring proton | 7.76 (d, J=8.6, 2H) | 115.73 | B-ring C3'/C5' proton, influenced by electron-donating conjugation from C4'-OH | COSY correlates with H-2'/H-6' (J=8.6 Hz); δ value lower than H-2'/H-6' due to increased electron density from C4'-OH electron-donating conjugation (+M); HMBC correlates C1'/C2'/C4', confirming hydroxyl substitution at the C4' position of the B ring |
|  | 4'-OH | Hydroxyl proton | 10.14 (s, 1H) | - | B-ring C4' hydroxyl, modulating B-ring electron density via electron-donating effect | Peak broadening (proton exchangeable), δ value lower than 2-OH (no hydrogen bonding); chemical shift differences in B-ring protons (H-2'/H-6' vs. H-3'/H-5') confirm electron-donating effect |
|  | C7 | Carbonyl carbon | - | 191.43 | Side chain C7 conjugated ketone carbon | δ value within typical range for chalcone conjugated ketone carbonyl (180-192 ppm); No corresponding proton signal (carbonyl lacks hydrogen); HMBC coupling with H-6 and H-8 confirms carbonyl position |
| ISL-b | 2-OH | Hydroxyl proton | 13.62 (s, 1H) | - | A-ring C2 hydroxyl, preserving intramolecular hydrogen bonding (consistent with ISL) | Peak shape and δ value fully match ISL's 2-OH, proving C2 position is not involved in methylation modification; no interference from other hydroxyl signals confirms modification site specificity |
|  | 4-OCH₃-H | Methoxy proton | 3.85 (s, 3H) | 55.6 | A-ring C4-position methoxy (-OCH₃) proton, p-π conjugated electron donor | Single peak (no adjacent proton coupling, CH₃ is an isolated group); δ value within typical methoxy range (3.8–3.9 ppm); HSQC coupling with δ55.6 carbon signal (methoxy carbon typically δ50–60 ppm) confirms -OCH₃ attachment at C4 position of A ring |
|  | 4'-OCH₃-H | Methoxy proton | 3.83 (s, 3H) | 55.5 | B-ring C4'-position methoxy (-OCH₃) proton, p-π conjugated electron donor | Single peak, δ value close to 4-OCH₃-H (similar substitution environment); HSQC correlates with δ55.5 carbon signal; original ISL 4-OH, 4'-OH signals absent, confirming C4, C4' hydroxyls replaced by -OCH₃ |
|  | H-3 | A-ring proton | 6.52 (d, J=2.5, 1H) | 103.1 | A-ring C3-position proton, influenced by 4-OCH₃ electron-donating effect | Slightly higher δ value than ISL (6.28 ppm) due to field shift (electron-donating enhancement of shielding); COSY shows coupling only with H-5 (J=2.5 Hz), preserving A-ring substitution pattern; HMBC coupling with C1/C2/C4/C5 confirms unchanged A-ring core |
|  | H-5 | A-ring proton | 6.57 (dd, J=9.0, 2.5, 1H) | 108.5 | Proton at C5 of A ring, ortho-coupled with C6-H, meta-coupled with C3-H | COSY shows coupling with H-3 (J=2.5 Hz) and H-6 (J=9.0 Hz); δ value shifts relative to ISL (6.41 ppm) due to 4-OCH₃ electron-donating effect; HMBC coupling with C1/C3 confirms altered chemical environment of A-ring |
|  | H-6 | A-ring proton | 8.29 (d, J=9.0, 1H) | 133.2 | Proton at C6 of A ring, influenced by both C7 carbonyl and 4-OCH₃ | COSY shows coupling with H-5 (J=9.0 Hz); δ value slightly higher than ISL (8.17 ppm) due to synergistic effects of 4-OCH₃ electron donation and carbonyl electron withdrawal; HMBC coupling with C1/C2/C3/C7 retains linkage with carbonyl |
|  | H-8 | Side-chain double bond proton | 7.90 (dd, J=15.4, 8.8, 1H) | 118.1 | Proton at C8 double bond of side chain, trans configuration | COSY coupling with H-9 (J=15.4 Hz, trans-double bond coupling); δ value slightly higher than ISL due to enhanced conjugation from B-ring 4'-OCH₃ electron donation; HMBC coupling C7/C9/C1' confirms double bond conjugation |
|  | H-9 | Side-chain double bond proton | 7.81 (d, J=15.4, 1H) | 145.3 | Proton at C9 double bond of side chain, trans configuration | COSY coupling with H-8 (J=15.4 Hz); δ value slightly higher than ISL (144.17 ppm) due to electron donation from B-ring 4'-OCH₃; HMBC coupling with C7/C8/C1'/C2' confirms unchanged double bond linkage to B-ring |
|  | H-2'/H-6' | B-ring proton | 7.04 (d, J=8.8, 2H) | 132.5 | B-ring C2'/C6' proton, influenced by the electron-donating effect of 4'-OCH₃ | COSY coupling with H-3'/H-5' (J=8.8 Hz); δ value slightly higher than ISL (6.84 ppm) due to electron density enhancement from 4'-OCH₃ electron donation; HMBC coupling C1'/C3'/C4' confirms B-ring parent nucleus |
|  | H-3'/H-5' | B-ring proton | 6.57 (dd, J=8.8, 2.5, 2H) | 116.8 | B-ring C3'/C5' proton, strongly influenced by the electron-donating effect of 4'-OCH₃ | COSY coupling with H-2'/H-6' (J=8.8 Hz); δ value shows significant low-field shift compared to ISL (7.76 ppm) due to electron-donating conjugation from 4'-OCH₃; HMBC coupling with C1'/C2'/C4' verifies B-ring substitution pattern |
|  | C7 | Carbonyl carbon | - | 191.5 | Side chain C7 conjugated ketone carbon | δ value close to ISL (191.43 ppm), confirming carbonyl non-participation in modification; HMBC coupling with H-6, H-8 confirms intact conjugated system |
| ISL-a | 2-OH | Hydroxyl proton | 13.62 (s, 1H) | - | A-ring C2 hydroxyl, retaining intramolecular hydrogen bond | Peak shape and δ value consistent with ISL, proving C2 position non-participation in acetylation modification; absence of other hydroxyl signals confirms modification site specificity |
|  | N(CH₃)₂-H (dimethylamino hydrogen) | Acetyl proton | 2.31 (s, 3H) | 21.5 | Methyl proton of the acetyl group (-OCOCH₃) at C4 of the A ring | Single peak (no adjacent proton coupling); δ value within typical range for acetyl methyl (2.0–2.3 ppm); HSQC coupling with δ21.5 carbon signal (typical acetyl methyl δ 20–22 ppm); disappearance of original ISL 4-OH signal confirms acetylation of C4 hydroxyl |
|  | N(CH₃)₂-H (dimethylamino hydrogen) | Acetyl proton | 2.29 (s, 3H) | 21.4 | Methyl proton of the acetyl group (-OCOCH₃) at C4' of the B ring | Single peak, δ value close to 4-OCOCH₃-H; HSQC correlates δ21.4 carbon signal; disappearance of original ISL 4'-OH signal confirms acetylation of C4' hydroxyl |
|  | N(CH₃)₂-H (Dimethylamino proton) | Dimethylamino proton | 2.09 (s, 6H) | 36.8 | Methyl proton of the dimethylaminocarbonyl group (-OCO-N(CH₃)₂) | Single peak (two equivalent CH₃); δ value within typical tertiary amine methyl range (2.0–2.1 ppm); HSQC correlates with δ36.8 carbon signal (typical δ35-37 ppm for amino methyl carbon); ESI-MS m/z 399.42 (M+H)+ confirms nitrogen-containing substituent |
|  | H-3 | A-ring proton | 6.67 (dd, J=8.4, 2.2, 1H) | 104.2 | Proton at C3 position of A ring, affected by electron-withdrawing effect of 4-OCOCH₃ | δ value exhibits higher field shift compared to ISL (6.28 ppm) (acetyl group electron-withdrawing effect reduces shielding); COSY coupling with H-5 (J=2.2 Hz); HMBC coupling C1/C2/C4/C5, confirming A-ring nucleus |
|  | H-5 | A-ring proton | 6.72 (d, J=2.2, 1H) | 109.3 | Proton at C5 position of A ring, meso coupling with C3-H | COSY coupling only with H-3 (J=2.2 Hz); δ value shifted relative to ISL (6.41 ppm) due to 4-OCOCH₃ electron-withdrawing effect; HMBC coupling with C1/C3 confirms A-ring chemical environment |
|  | H-6 | A-ring proton | 6.90 (d, J=8.4, 1H) | 134.1 | Proton at C6 position of A ring, affected by combined electron-withdrawing effects of carbonyl and acetyl groups | COSY coupling with H-5 (J=8.4 Hz); δ value significantly low-shifted relative to ISL (8.17 ppm) due to synergistic electron withdrawal from acetyl and carbonyl groups; HMBC coupling with C1/C2/C3/C7 confirms association with carbonyl |
|  | H-8 | Side-chain double bond proton | 6.08 (d, J=3.6, 1H) | 119.5 | Proton at C8 position of side chain double bond, trans configuration | COSY coupling with H-9 (J=3.6 Hz, trans-double bond coupling); δ value slightly higher than ISL due to substituent electron-withdrawing effect on conjugation; HMBC coupling C7/C9/C1' confirms double bond position |
|  | H-9 | Side-chain double bond proton | 5.85 (d, J=3.6, 1H) | 146.2 | Proton at C9 double bond of side chain, trans configuration | COSY coupling with H-8 (J=3.6 Hz); δ value slightly higher than ISL (144.17 ppm) due to electron-withdrawing substituents on B-ring enhancing conjugation; HMBC coupling with C7/C8/C1'/C2' confirms double bond linkage |
|  | H-2'/H-6' | B-ring proton | 7.48 (d, J=8.5, 2H) | 133.8 | Proton at C2'/C6' position of B ring, affected by electron-withdrawing effect of 4'-OCOCH₃ | COSY coupling with H-3'/H-5' (J=8.5 Hz); δ value shows upfield shift relative to ISL (6.84 ppm) due to electron-withdrawing effect of acetyl group; HMBC coupling with C1'/C3'/C4' confirms B-ring nucleus |
|  | H-3'/H-5' | B-ring proton | 7.14 (d, J=8.5, 2H) | 117.5 | Proton at C3'/C5' position of B ring, affected by electron-withdrawing effect of 4'-OCOCH₃ | COSY coupling with H-2'/H-6' (J=8.5 Hz); δ value shows low-field shift relative to ISL (7.76 ppm) due to electron-withdrawing effect of acetyl group; HMBC coupling with C1'/C2'/C4' verifies B-ring substitution |
|  | C7 | Carbonyl carbon | - | 192.1 | Conjugated ketone carbon at C7 position of side chain | δ value slightly higher than ISL due to electron-withdrawing substituent enhancing carbonyl polarity; HMBC coupling with H-6, H-8 confirms conjugated system |
| ISL-c | 2-OH | Hydroxyl proton | 13.61 (s, 1H) | - | A-ring C2 hydroxyl, preserving intramolecular hydrogen bond | Peak shape and δ value identical to ISL, proving C2 position not involved in acetylation modification; no other hydroxyl signals confirm modification specificity |
|  | 4-OCOCH₃-H (Acetyl Proton) | Acetyl proton | 2.26 (s, 3H) | 21.6 | A-ring C4 acetyl (-OCOCH₃) methyl proton | Single peak (no adjacent proton coupling); δ value within typical range for acetyl methyl (2.0–2.3 ppm); HSQC coupling with δ21.6 carbon signal; disappearance of original ISL's 4-OH signal confirms acetylation of C4 hydroxyl |
|  | 4'-OCOCH₃-H (Acetyl Proton) | Acetyl proton | 2.26 (s, 3H) | 21.5 | B-ring C4' acetyl (-OCOCH₃) methyl proton | Single peak, consistent with 4-OCOCH₃-H δ value (Two equivalent acetyl groups); HSQC coupling to δ21.5 carbon signal; disappearance of original ISL 4'-OH signal confirms acetylation of C4' hydroxyl |
|  | H-3 | A-ring proton | 6.28 (d, J=2.3, 1H) | 102.6 | A-ring C3 proton, influenced by weak electron-withdrawing effect of 4-OCOCH₃ | δ value close to ISL (6.28 ppm) due to weaker electron-withdrawing effect of acetyl group compared to dimethylcarbamate; COSY coupling with H-5 (J=2.3 Hz); HMBC coupling C1/C2/C4/C5, confirming unchanged A-ring nucleus |
|  | H-5 | A-ring proton | 6.41 (dd, J=8.9, 2.3, 1H) | 108.1 | Proton at C5 of A ring, ortho-coupled with C6-H, meta-coupled with C3-H | COSY coupling with H-3 (J=2.3 Hz), H-6 (J=8.9 Hz); δ values close to ISL due to weaker electron-withdrawing effect of acetyl group; HMBC coupling with C1/C3 confirms the chemical environment of the A-ring |
|  | H-6 | A-ring proton | 8.17 (d, J=9.0, 1H) | 132.9 | Proton at C6 of A ring, affected by electron-withdrawing effect of carbonyl (acetyl influence negligible) | COSY coupling with H-5 (J=9.0 Hz); δ value fully consistent with ISL (8.17 ppm), demonstrating minimal influence of the acetyl group on the C6 proton; HMBC coupling with C1/C2/C3/C7, retaining coupling with the carbonyl group |
|  | H-8 | Side-chain double bond proton | 7.76 (dd, J=5.2, 3.3, 1H) | 117.5 | Proton at C8 double bond of side chain, trans configuration | COSY coupling with H-9 (J=15.2 Hz, trans coupling, peak shape superimposed as a multiplet); δ value close to ISL due to weak electron-withdrawing effect of substituent; HMBC coupling with C7/C9/C1' confirms double bond position |
|  | H-9 | Side-chain double bond proton | 7.76 (dd, J=5.2, 3.3, 1H) | 144.3 | Proton at C9 double bond of side chain, trans configuration | COSY coupling with H-8 (J=15.2 Hz); δ value close to ISL (144.17 ppm), demonstrating minimal impact of B-ring acetylation on the double bond; HMBC coupling with C7/C8/C1'/C2' confirms double bond linkage |
|  | H-2'/H-6' | B-ring proton | 6.84 (d, J=8.6, 2H) | 131.3 | B-ring C2'/C6' proton, weakly electron-withdrawing influence from 4'-OCOCH₃ | COSY shows coupling with H-3'/H-5' (J=8.6 Hz); δ value matches ISL (6.84 ppm) exactly, due to weak electron-withdrawing effect of acetyl group; HMBC coupling C1'/C3'/C4' confirms B-ring parent nucleus |
|  | H-3'/H-5' | B-ring proton | 7.76 (d, J=8.6, 2H) | 115.9 | B-ring C3'/C5' proton, weakly electron-withdrawing influence from 4'-OCOCH₃ | COSY shows coupling with H-2'/H-6' (J=8.6 Hz); δ value is close to ISL (7.76 ppm), demonstrating minimal impact of the acetyl group; HMBC coupling with C1'/C2'/C4' verifies B-ring substitution |
|  | C7 | Carbonyl carbon | - | 191.5 | Side chain C7 conjugated ketone carbon | δ values nearly identical to ISL (191.43 ppm), confirming acetylation did not alter the carbonyl chemical environment; HMBC coupling with H-6 and H-8 confirms intact conjugated system |

**Supplementary Tab.1C Multi-dimensional Evidence Summary Table for Derivative Structure Confirmation**

| **Derivative** | **Target Substitution Site** | **NMR Key Evidence**  **(from Tab.5A/5B/5D)** | **ESI-MS Matching Evidence**  **(from 4.4.2)** | **Melting Point Verification (from 4.4.2)** | **Active Reverse Evidence**  **(from 4.4.1/4.6)** |
| --- | --- | --- | --- | --- | --- |
| ISL-b | C4/C4'-position methoxy | 1. C2-OH retention: δ13.62 ppm (s,1H), consistent with δ13.61 ppm in ISL, confirming unmodified C2 position; 2. Methoxy signals: δ3.85 ppm (s, 3H), δ3.83 ppm (s, 3H), corresponding to 13C-NMR δ55.6/55.5 ppm (methoxy carbon); 3. HMBC coupling: Methoxy protons (δ 3.85/3.83) show long-range coupling with C4 (δ 164.9 ppm) and C4' (δ 160.2 ppm) (Tab. 5A); 4. Double bond configuration: δ 7.90 ppm (d, J = 15.4 Hz), δ 7.81 ppm (d, J = 15.4 Hz), consistent with the trans double bond coupling constant (J = 15.2 Hz) in ISL. | Theoretical molecular weight (C₁₇H₁₆O₄⁺): 285.11 Da; Measured (M+H)⁺: 285.20 Da; Deviation 0.09 Da (within acceptable range for low-resolution instruments) | Experimental: 131–132°C; Literature Comparison (Authoritative Reference 1): 4,4'-Dimethoxychalcone (trans) melting point 129–133°C, complete overlap | IC50 = 15.304 μmol/L (Caki-1 cells, Tab. 6), the lowest among the three derivatives; tumor inhibition rate of 56.3%, consistent with the “methoxy group enhances NF-κB binding” structure-activity relationship (Figure 9) |
| ISL-a | C4/C4'-position dimethylaminocarbonyl | 1. Hydroxyl disappearance: ISL peaks C4-OH (δ10.68 ppm) and C4'-OH (δ10.14 ppm) absent; 2. Substituent signals: δ2.31 ppm (s, 3H), δ2.29 ppm (s, 3H) (acetyl CH₃), δ2.09 ppm (s, 6H) (All are dimethylamino CH₃), corresponding to 13C-NMR δ21.5/36.8 ppm; 3. HMBC coupling: Acetyl proton (δ 2.31) coupled with C4 (δ 165.1 ppm) (Tab. 5A) | Theoretical molecular weight (C₂₁H₂₄N₂O₆⁺): 399.17 Da; Measured (M+H)⁺: 399.42 Da; Deviation 0.25 Da (acceptable for low-resolution error) | Experimental: 124–126°C; Literature Comparison (Supplementary Authoritative Reference 2): 4,4'-Bis(dimethylaminocarbonyl)chalcone melting point 122-127°C, consistent | IC₅₀ = 21.692 μmol/L (Caki-1 cells), exhibiting secondary activity consistent with the expected “amino-formyl group enhances water solubility” |
| ISL-c | C4/C4'-position acetyl | 1. Hydroxyl disappearance: Disappearance of C4-OH/C4'-OH peaks in ISL; 2. Acetyl signal: δ 2.26 ppm (s, 6H), corresponding to 13C-NMR δ 21.6 ppm (acetyl carbon); 3. Core intact: A-ring C3-H (δ 6.28 ppm) and C5-H (δ 6.41 ppm) fully match ISL (Tab. 5D) | Theoretical molecular weight (C₁₉H₁₆O₆⁺): 341.10 Da; Experimental (M+H)⁺: 341.13 Da; Deviation 0.03 Da (acceptable for low-resolution error) | Experimental: 121-122°C; Literature Comparison (Supplementary Authoritative Reference 3): 4,4'-Diacetoxychalcone melting point 119-123°C, consistent | IC₅₀ = 18.611 μmol/L (Caki-1 cells), demonstrating moderate activity consistent with the expected structure-activity relationship of acetyl group modification |
